# Built-in selection or confounder bias? *Dynamic Landmarking* in matched propensity score analyses

**DOI:** 10.1186/s12874-024-02444-7

**Published:** 2024-12-21

**Authors:** Alexandra Strobel, Andreas Wienke, Jan Gummert, Sabine Bleiziffer, Oliver Kuss

**Affiliations:** 1 Institute of Medical Epidemiology, Biostatistics, and Informatics, Interdisciplinary Center for Health Sciences, Medical Faculty, Martin-Luther-University Halle Wittenberg, Halle, Germany; 2 Heart and Diabetes Center North Rhine-Westphalia, Ruhr-University Bochum, Bad Oeynhausen, Germany; 3 German Diabetes Center, Leibniz Center for Diabetes Research, Institute for Biometrics and Epidemiology, Heinrich-Heine-University Düsseldorf, Düsseldorf, Germany; 4 Centre for Health and Society, Faculty of Medicine, Heinrich-Heine-University Düsseldorf, Düsseldorf, Germany

**Keywords:** Cox model, Hazard ratio, Built-in selection bias, Confounding bias

## Abstract

**Background:**

Propensity score matching has become a popular method for estimating causal treatment effects in non-randomized studies. However, for time-to-event outcomes, the estimation of hazard ratios based on propensity scores can be challenging if omitted or unobserved covariates are present. Not accounting for such covariates could lead to treatment estimates, differing from the estimate of interest. However, researchers often do not know whether (and, if so, which) covariates will cause this divergence.

**Methods:**

To address this issue, we extended a previously described method, *Dynamic Landmarking*, which was originally developed for randomized trials. The method is based on successively deletion of sorted observations and gradually fitting univariable Cox models. In addition, the balance of observed, but omitted covariates can be measured by the sum of squared z-differences.

**Results:**

By simulation we show, that *Dynamic Landmarking* provides a good visual tool for detecting and distinguishing treatment effect estimates underlying built-in selection or confounding bias. We illustrate the approach with a data set from cardiac surgery and provide some recommendations on how to use and interpret *Dynamic Landmarking* in propensity score matched studies.

**Conclusion:**

*Dynamic Landmarking* is a useful post-hoc diagnosis tool for visualizing whether an estimated hazard ratio could be distorted by confounding or built-in selection bias.

**Supplementary Information:**

The online version contains supplementary material available at 10.1186/s12874-024-02444-7.

## Background

Randomized controlled trials (RCTs) are the gold standard for evaluating treatment effects in medical research, because random treatment allocation should guarantee balanced known and unknown covariates in the compared groups, resulting in the absence of confounding (for terminology used in manuscript see Tab. S1). However, even if confounding is minimized after randomization, prognostic factors (i.e. covariates that are associated with the outcome but not with treatment allocation) may still be present. For time-to-event data, the Cox model [7, 8] is commonly used for statistical analysis, providing the hazard ratio as the generic effect measure. Typically, in RCTs the Cox model does not include prognostic factors as covariates. Instead, a marginal Cox model with only the treatment as a single covariate is estimated, yielding a marginal hazard ratio that is interpreted as a population-averaged treatment effect. However, there is often interest in understanding treatment effects at a subject-specific level. A subject-specific (conditional) interpretation of the hazard ratio can only be made when conditioning the Cox model on all prognostic factors. This particularly means that if a single prognostic factor (whether observed or unobserved) is omitted from the Cox model, it would prevent the hazard ratio from being interpreted on a subject-specific level. More precise, assume a proportional hazards model (1)

1 $$\:\lambda\:\left(t|Z,U\right)=\:{\lambda\:}_{0}\left(t\right)\text{e}\text{x}\text{p}({\beta\:}_{Z}Z+\:{\beta\:}_{U}U)\:\:\:$$

where $\:{\lambda\:}_{0}\left(t\right)$ is an unspecified baseline hazard function, depending on time $\:t$ and is assumed to be common across all individuals. Furthermore, $\:Z$ and $\:U\:$are some observed covariates with their corresponding regression coefficients $\:{\beta\:}_{Z}$ and$\:\:{\beta\:}_{U}$. Then $\:\lambda\:\left(t\right|Z,U)\:$defines the conditional hazard with $\:{\beta\:}_{Z}$ summarizing the conditional effect of$\:\:Z$, yielding a subject-specific interpretation. On the other hand, if $\:U$ will be omitted, one would estimate model (2), i.e.:

2 $$\:\lambda\:\left(t|Z\right)={\lambda\:}_{0}\left(t\right)\text{exp}\left({\beta\:}_{Z}Z\right)$$

with $\:\lambda\:\left(t\right|Z)$ reflecting the marginal hazard, yielding an population-averaged interpretation. Importantly, conditional and marginal Cox models will not provide the same estimates for a treatment effect if additional prognostic factors are associated with the time-to-event outcome [9, 29, 30]. This circumstance is referred to as “non-collapsibility”, indicating that the magnitude of the effect measure is changing when conditioning on a prognostic factor [10]. This is often accompanied by the term “built-in selection bias”, which can be seen as result of conditioning on previous survival within hazard rates. More precise, assume an omitted prognostic factors (i.e., measured during the trial but omitted from the Cox model), which introduces heterogeneity, causing individuals at higher baseline risk (regarding omitted prognostic factors) to expect the event earlier than those at lower risk [1, 17]. Given an effective treatment, this would result in higher-risk individuals surviving longer in the treated group than in the control group. This results in a deviation from the marginal and conditional hazard ratio, due to conditioning on prior survival. Depending on the magnitude of the treatment effect, the influence of the omitted prognostic factor on the time-to-event outcome and the follow-up time, the magnitude of the built-in selection bias changes [5, 28, 31]. Therefore, when aiming for a conditional treatment effect (more precise, conditional on all prognostic factors) in RCTs, all prognostic factors have to be included in the Cox model. Please note: In the case where treatment is the only prognostic factor influencing time-to-event and there are no other prognostic factors, the marginal model and the conditional model would give the same value for the marginal and the conditional hazard ratio. This is because the Cox model would then include all relevant prognostic factors, that is, only the treatment allocation, and no other adjustments are needed for estimating a conditional treatment effect. As a result, non-collapsibility would not be an issue and thus built-in selection bias would not occur.

In non-randomized trails, the situation might be more complex because confounding becomes an additional issue. Here, treatment allocation is generally determined by baseline characteristics, leading to systematic differences between treatment groups [25]. One prominent way to address these baseline differences is balancing the data by Propensity Score (PS) matching [26, 27]. Here, in a first step the PS for each individual is usually estimated via a logistic regression model. In a second step the PS is used for estimating the treatment effect of interest (that is, in our case the hazard ratio) [21]. Under the assumptions of positivity, consistency, and unconfoundedness for the PS, valid causal statements about treatment effects can be made. Misspecification of the PS model due to the omission of relevant confounders would lead to confounding bias, resulting in a biased treatment effect estimate. However, even if the PS model includes all confounders, non-collapsibility (and the corresponding built-in selection bias) plays a role when fitting a Cox model in the PS matched trial. Usually, as in RCTs, a marginal Cox model with the treatment effect as the single covariate is fitted to the data, yielding a marginal (population-averaged) treatment effect estimate. However, when aiming for a conditional (subject-specific) treatment effect, the Cox model needs to be conditional on all relevant prognostic factors. Note that prognostic factors cannot be taken into account by PS models, as the PS addresses the association between a covariate and the treatment allocation, which (by definition) is not present in prognostic factors. Therefore, when estimating a treatment effect in PS matched trials, two potential issues could arise when covariates are omitted from the analysis. First, omitting a prognostic factor from the Cox model would lead to the built-in selection bias. Second, omitting a confounder from the PS model would entail confounding bias. Both issues have the consequence that the final treatment effect estimate differs from the estimate of interest (that is, a conditional and unbiased treatment effect) [6, 14]. For an overview of concepts and comparison in RCTs and PS-matched trials please see Tab. S2.

The choice of covariates for the PS model and the subsequent outcome model relies on scientific understanding and clinical expertise. This especially introduces the possibility of omission of covariates that were measured during the trial, but not included in the PS model or, after PS matching, in the Cox model. It is therefore of interest to investigate whether an estimated treatment effect is subject to confounding bias or built-in selection bias. Unfortunately, the hazard ratio provides the effect in a single number, not giving a hint for any of these issues. Therefore, a recent article introduced a new method, *Dynamic Landmarking*, for diagnosing whether an estimated treatment effect from a Cox model was subject to built-in selection bias in RCTs [32]. The original methodological approach was designed to detect potential prognostic factors that are measured but omitted from the Cox model and could therefore induce built-in selection bias.

The aim of the present work is to extend the existing *Dynamic Landmarking* approach to PS matched trials. More precisely, we want to use *Dynamic Landmarking* as a post-hoc diagnosing tool in order to check if the estimated hazard ratio could be distorted by confounding or built-in selection bias. Moreover, we are interested in detecting covariates that were observed (e.g., are present in the data set), but omitted from the analysis, which could either induce potential built-in selection or confounding bias.

First, we describe the extension of *Dynamic Landmarking* to the PS matched case. Second, we give the results of a simulation study to examine how the approach performs in a PS matched trial. Third, we apply the extended procedure to a real data set from cardiac surgery and finally discuss the results.

## Methods

The original *Dynamic Landmarking* is a methodological approach, which provides a visual tool for diagnosing if an estimated treatment effect is subject to built-in selection bias. Furthermore, omitted prognostic factors that are measured during the trial but omitted from the Cox model, are investigated whether they induce built-in selection bias. The idea of *Dynamic Landmarking* is quite simple: First, the dataset is sorted by observation time and a univariable Cox model only including the treatment is fitted to the full data set. Afterwards, the earliest $\:M\:\left(M>0\right)$ observations are deleted regardless of the event status (observed or censored) and a new univariable Cox model is fitted to the smaller data set. After each deletion step, the start of the follow-up interval for the new Cox model is moved forwards. More precisely, the new time zero for the new Cox model corresponds to the follow-up time of the latest of the $\:M\:$deleted individuals in the previous step. This procedure of deleting the earliest $\:M\:$observations and refitting univariable Cox models is continued until the data set no longer contains a sufficient number of observations for convergence. In general, high-risk individuals will have shorter observation times than low-risk individuals, as they tend to expect the event of interest earlier. Consequently, individuals with higher baseline risk (regarding the omitted prognostic factors) will be deleted earlier during *Dynamic Landmarking*.

In parallel, the balance of omitted prognostic factors is measured in each step by the sum of squared z-differences ($\:SS{Q}_{zDiff}$) [19], with $\:SS{Q}_{zDiff}=\sum\:{z}_{con}^{2}+\sum\:{z}_{bin}^{2}+\sum\:{z}_{ord}^{2}+\sum\:{z}_{nom}^{2}\:$, whereby e.g.,

$$\:{z}_{cont}=\frac{{\stackrel{-}{x}}_{T}-\:{\stackrel{-}{x}}_{C}}{\sqrt{\frac{{\widehat{\sigma\:}}_{T}^{2}}{{N}_{T}}+\frac{{\widehat{\sigma\:}}_{C}^{2}}{{N}_{C}}}}\:\:\:\:\:\:\:\:\:\:\text{a}\text{n}\text{d}\:\:\:\:\:\:\:\:{z}_{bin}=\frac{{\widehat{p}}_{T}-{\widehat{p}}_{C}}{\sqrt{\frac{{\widehat{p}}_{T}(1-{\widehat{p}}_{T})}{{N}_{T}}+\frac{{\widehat{p}}_{C}(1-{\widehat{p}}_{C})}{{N}_{C}}}}\:.$$

Here $\:{\stackrel{-}{x}}_{T},\:{\stackrel{-}{x}}_{C},\:{\widehat{\sigma\:}}_{T}^{2},\:{\widehat{\sigma\:}}_{C}^{\:2},\:{\widehat{p}}_{T},\:{\widehat{p}}_{C},\:{N}_{T},\:{N}_{C}\:$denote the respective estimated means, variances, proportions, and sample sizes of the two groups (formula for all z-differences can be found in Formula S1). The $\:SS{Q}_{zDiff}$ is a global balance measure and follows a chi-squared-distribution with expectation $\:k$ for $\:k$ independent covariates.

After each deletion-and-refitting step, the point estimator for the treatment effect and the$\:\:SS{Q}_{zDiff}$ is saved, yielding a trajectory depending on the remaining number of individuals. Through the systematic removal of individuals, treatment effects are gradually estimated within a population of lower-risk patients, potentially leading to a systematic shift in the effect trajectory due to the presence of built-in selection bias. Moreover, a potential imbalance in omitted prognostic factors arises, manifesting as a systematic shift in the $\:SS{Q}_{zDiff}\:$trajectory [32].

To apply *Dynamic Landmarking* in non-randomized trials, a balancing procedure, e.g. PS matching, has to be applied prior to sorting the data regarding the observation time. Afterward, the original *Dynamic Landmarking* is carried out. However, note that omitted variables in RCTs (by design) can only be prognostic factors. In PS matched studies, however, they can be both prognostic factors and confounders. This potentially creates two problems, first built-in selection bias due to omission of prognostic factors and, second, confounding bias due to omitted confounders, and of course, both should be addressed separately by *Dynamic Landmarking*. This distinction between omitted prognostic factors and omitted confounders can be made by looking at the definition of $\:SS{Q}_{zDiff}$: Omitting a observed confounder from the PS model would result in unbalanced groups after PS matching. This is because the association of the omitted confounder with the treatment allocation is still present, resulting in large values of $\:SS{Q}_{zDiff}$ already at the beginning of *Dynamic Landmarking*, that is, before the first deletion step. Omitting a prognostic factor from the Cox model on the other hand would still yield balanced groups after PS matching resulting in lower initial values of$\:\:SS{Q}_{zDiff}$. Hence, initial $\:SS{Q}_{zDiff}\:$-values for the full data set will give a first hint on whether the omitted variable is a confounder or a prognostic factor.

The following preconditions must be met in order to achieve valid results from *Dynamic Landmarking*: First, independent censoring has to be assumed. Second, the conditional hazard ratio for treatment is assumed to be constant across the population and over time, i.e. proportional hazards hold and treatment effect is time-invariant. Third, for measuring the balance by $\:SS{Q}_{zDiff}\,$at least one available covariate has to be omitted from either the PS or the Cox model.

## Results from a simulation study

### Data generation process

**Fig. 1:**
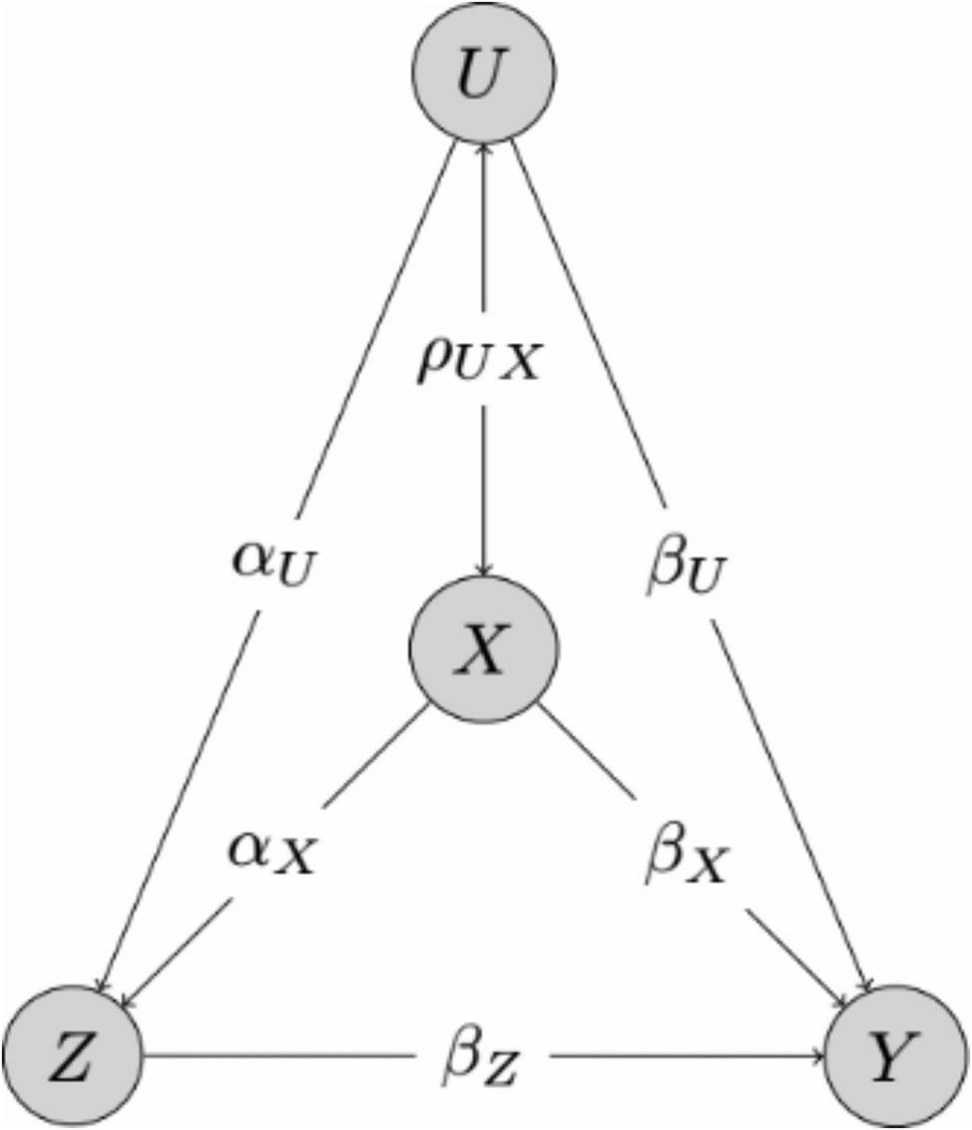
Graphical illustration for data generation process

We simulated a non-randomized intervention trial with $\:Z$ denoting the treatment, $\:Y$ the time-to-event outcome, $\:X$ a known and measured confounder and $\:U$ an omitted covariate, see Fig. 1 for the corresponding graphical illustration of the data generation process. Both, $\:X$ and$\:\:U$, follow a standard normal distribution. First, we simulated the probability of treatment allocation for each subject $\:i\:$from the logistic model

$$\:logit\left({p}_{i}\right)={\alpha\:}_{0}+{\alpha\:}_{X}\cdot\:{X}_{i}+\:{\alpha\:}_{U}\cdot\:{U}_{i}.$$

For the intercept, $\:{\alpha\:}_{0}=\:-1.21\:$was chosen in order to obtain approximately 24% treated individuals, which was motivated by the empirical example in Section “Illustration of the procedure with an example from cardiac surgery”. The parameter $\:{\alpha\:}_{X}\:$was set to $\text{log}\left(3\right)$. This denotes a strong impact of the confounder $\:X\:$on the treatment assignment. Afterwards, we generated the actual treatment status $\:{Z}_{i}\:$from a Bernoulli distribution with subject-specific probability$\:{\:p}_{i}$. We then simulated the time-to-event outcome $\:{Y}_{i}\:$for each individual using a Weibull baseline hazard with parameters$\:\:\lambda\:=0.01$ and$\:\:\gamma\:=1.5$. The final hazard function used was:

$$\:h\left(t|Z,X,U\right)=\gamma\:\lambda\:{t}^{\gamma\:-1}\cdot\:{e}^{{\beta\:}_{Z}Z+{\beta\:}_{X}X+{\beta\:}_{u}U}.$$

For the regression parameter$$\:\:{\beta\:}_{X}$$ we used the value$$\:\text{log}\left(3\right)\:$$, which was intended to denote a strong impact of $$\:X$$ on the time-to-event outcome. We considered different effects of $$\:U$$ on treatment allocation ($$\eqalign{& \>\alpha {\>_U} \in \>\{ {\rm{log}}\left( {0.5} \right),{\rm{log}}\left( {0.66} \right),{\rm{log}}\left( {0.8} \right), \cr & \,\,\,\,\,\,\,\,\,\,\,\,\,\>{\rm{log}}\left( 1 \right),{\rm{log}}\left( {1.25} \right),\>{\rm{log}}\left( 2 \right),{\rm{log}}\left( 3 \right)\} \cr}$$). We further varied the effect of $$\:U$$ on the time-to-event outcome by using the following regression coefficients: $$\eqalign{& \>\beta {\>_U} \in \{ {\rm{log}}\left( {0.5} \right),{\rm{log}}\left( {0.66} \right),{\rm{log}}\left( {0.8} \right),\>{\rm{log}}\left( 1 \right), \cr & \,\,\,\,\,\,\,\,\,\,\,\,\,\,\,{\rm{log}}\left( {1.25} \right),{\rm{log}}\left( {1.5} \right),{\rm{log}}\left( 2 \right),{\rm{log}}\left( 3 \right)\} \>.\>\> \cr}$$Furthermore, we assumed various correlations between $$\:U$$ and$$\:\:X$$:$$\:{\:\rho\:}_{XU}\in\:\left\{0,\:\:0.2,\:\:0.6,\:\:0.9\right\}$$. Moreover, we considered different values for the conditional treatment effect: $$\:{\beta\:}_{Z}\in\:\{\text{log}\left(1.25\right),\text{l}\text{o}\text{g}(1.5),\text{l}\text{o}\text{g}(2),\text{l}\text{o}\text{g}(3\left)\right\}$$ and assumed censoring proportions of approximately 10%, 40% and 80% which were generated using a exponential distribution with parameter $$\lambda\:\in\:\{\text{0.2,0.6},\:0.9\}$$For each scenario, we simulated 500 data sets with 5,000 individuals each. Please be aware that $$\:U$$ is classified differently based on the values of $$\:{\alpha\:}_{U}\:$$and$$\:{\:\beta\:}_{U}\:$$$$\:U\:$$is considered an independent covariate when both $$\:{\alpha\:}_{U}=0\:$$and$$\:\:{\beta\:}_{U}=0\,$$ a prognostic factor when $$\:{\alpha\:}_{U}=0\:$$and$$\:{\:\beta\:}_{U}\ne\:0$$, an instrumental variable when $$\:{\alpha\:}_{U}\ne\:0\:$$and$$\:\:{\beta\:}_{U}=0$$, and finally, a confounder when both$$\:{\alpha\:}_{U}\ne\:0$$ and$$\:{\:\beta\:}_{U}\ne\:0$$.

### Data analyses

For each scenario, we estimated the PS by logistic regression, including the known confounder$\:\:X$, but excluding the covariate$\:\:U$: $\:logit\left({p}_{i}\right)=\:{\alpha\:}_{0}+\:{\alpha\:}_{X}\cdot\:{X}_{i}$. We then performed a 1:1 PS-matching without replacement. Each treated individual was matched with the greedy nearest available neighbour with a caliper width of 0.2 of the standard deviation of the logit of the propensity score [2, 3]. In a second step, we applied *Dynamic Landmarking* to the PS-matched data set. Therefore, we fitted stratified (for the matching stratum) Cox models with treatment as the only covariate:

3 $$\:{h}_{j}\left(t|Z\right)={h}_{0,j}\left(t\right)\cdot\:{e}^{{\beta\:}_{Z}Z}$$

Here, $\:{h}_{0,j}$ refers to the baseline hazard function for matching stratum$\:\:j$. These stratified (for matching stratum) Cox model will be referred to “stratified Cox model” from now on. Please note, that $\:U$ was omitted from both, the PS model and the Cox model, whereas $\:X$ was considered in the PS model in each scenario.

### Results

#### Omitting a prognostic factor – detecting induced built-in selection bias

**Fig. 2:**
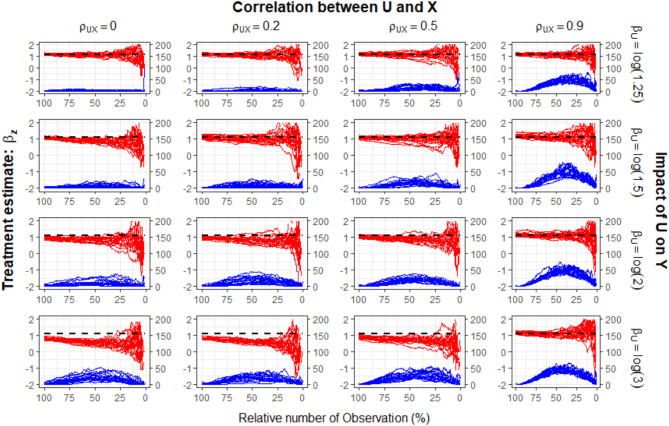
Trajectories of treatment effect (left y-axis, red) on the log(HR) scale and sum of squared z-differences (right y-axis, blue) for balance measuring of the omitted covariate $\:U$ for 500 simulated data sets. Dashed black lines show the true, simulated conditional treatment effect estimate$\:\:{\beta\:}_{Z}=\text{\:log}\left(3\right)$. All scenarios assume the omission of a prognostic factor $\:U$, i.e. $\:{\alpha\:}_{U}=0$., and a censoring rate of 10%

In Fig. 2 we give the results for an omitted prognostic factor $\:U$ (i.e.,$\:\:{a}_{U}=0)$, a highly effective treatment ($\:{\beta\:}_{Z}=\text{log}\left(3\right)$) and a censoring proportion of 10%. Results for smaller treatment effects and higher censoring proportions are given in the supplementary information (see Fig. S1 – Fig. S5). Two important things should be noted: First, in these scenarios, the PS model was correctly specified and built-in selection bias is induced by the omission of a prognostic factor. Second, the treatment effect trajectory will not be equal to the true simulated effect $\:\:{\beta\:}_{Z}\:$at the beginning of *Dynamic Landmarking*. This is because we show the percentage of remaining individuals on the x-axis and not the original observation time. As a result, the initial treatment effect estimate derived from *Dynamic Landmarking* corresponds to the estimate one would obtain at the end of a study using a stratified Cox model. However, since a relevant prognostic factor has been excluded, this initial estimate is already subject to built-in selection bias, leading to a discrepancy between the estimated and the true simulated effect from the beginning on.

The mean sample size of the PS matched data was 2,402 in the simulation. In the first column of Fig. 2, $\:U$ is independent of the confounder $\:X$ ($\:{\rho\:}_{UX}=\:0$). We observe that a higher impact of $\:U$ on the time-to-event outcome causes a more visible systematic shift in the treatment effect trajectory. Additionally, all scenarios show low initial $\:SS{Q}_{zDiff}$-values indicating the omission of a prognostic factor that is still balanced between the treatment groups after PS matching. Moreover an increase of the $\:SS{Q}_{zDiff}$-trajectory is observed during the deletion of the first 50% of observations. Similar results were obtained for smaller treatment effects and higher censoring rates. However, as highlighted by serveral authors [e.g. 31, 35], the built-in selection bias occurs less prominent in case of smaller treatment effects and smaller prognostic effects. Consequently, in such cases, Dynamic Landmarking would identify a less pronounced decline in treatment effect trajectories. In the remaining columns, we simulated a non-zero correlation between $\:X$ and $\:U$ varying it from weak to strong. Here we find that the estimated treatment effect moves closer to the true simulated one if the correlation gets stronger. Importantly, less systematic changes in the treatment effect trajectory can be observed. This is because the omitted prognostic factor $\:U$ is indirectly accounted for by including $\:X$ in the PS model, allowing a correction towards the true treatment effect. And of course, the stronger the correlation, the closer will the estimated hazard ratio be to the true, simulated one [14].

#### Omitting a confounder – detecting confounding bias

**Fig. 3:**
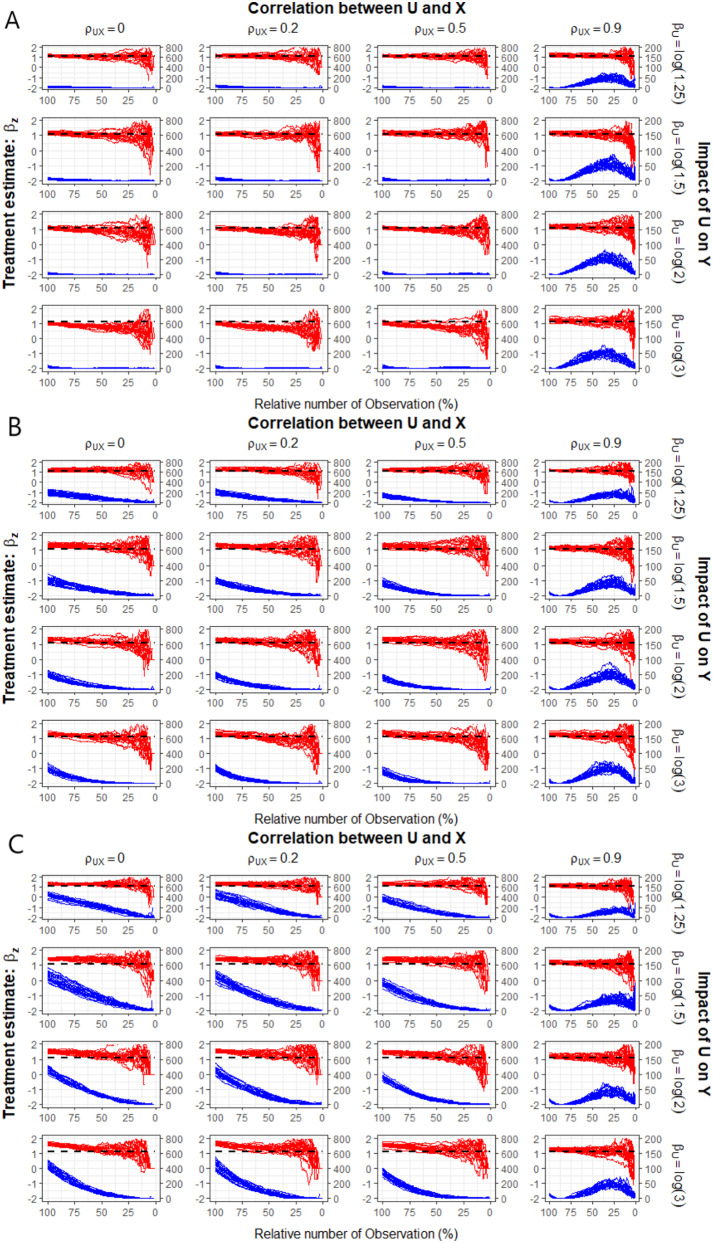
Trajectories of treatment effect (left y-axis, red) on the log(HR) scale and sum of squared z-differences (right y-axis, blue) for balance measuring of the omitted covariate $\:U$ for 500 simulated data sets. Dashed black lines show the true, conditional treatment estimate$\:\:{{\upbeta\:}}_{\text{z}}=\text{log}\left(3\right)$. All scenarios assume the omission of a true confounder $\:U$ with **A**: low impact on treatment allocation, i.e., $\:{\alpha\:}_{u}=\text{l}\text{o}\text{g}\left(1.25\right)$**B**: moderate impact on treatment allocation, i.e., $\:{\alpha\:}_{U}=\text{l}\text{o}\text{g}\left(2\right)$. **C**: high impact on treatment allocation, i.e. $\:{\alpha\:}_{U}=\text{l}\text{o}\text{g}\left(3\right)$

The results of the simulation when omitting a true confounder (i.e., $\:{\alpha\:}_{U}\ne\:0$) from the PS model are shown in Fig. 3. We present the results for a true, simulated treatment effect of $\:{\beta\:}_{Z}=\text{l}\text{o}\text{g}\left(3\right)$ and a censoring proportion of 10%. Results for smaller treatment effects can be found in the supplementary material (see Fig. S5 and Fig. S6). Moreover, negative values of $\:{\alpha\:}_{U}$ and $\:{\beta\:}_{U}$ (and combinations) are considered in Fig. S8 - Fig. S10. Note, that all these scenarios cover the case when the PS model is missspecified as a relevant confounder is omitted. In addition, there are no (omitted) prognostic factors simulated in this scenario. In the first column, we again assume that an independent confounder has been omitted ($\:{\rho\:}_{UX}=0$). As in the first simulation (Section “Omitting a prognostic factor – Detecting induced built-in selection bias”), we observe a more visible systematic shift in the trajectory of the treatment effects while the influence of $\:U$ on the time-to-event outcome increases. Moreover, the systematic shift can be observed more clearly when the omitted confounder is strongly associated with treatment allocation (see the first column of Fig. 3A compared to first column of Fig. 3B and C). In other words, *Dynamic Landmarking* better detects confounding bias if the association with the treatment allocation is strong (i.e., $\:\left|{\alpha\:}_{u}\right|\gg\:0)$. The $\:SS{Q}_{zDiff}$-trajectories behave in an expected way, i.e., achieving extremely high values at the beginning of *Dynamic Landmarking*. Referring to the formula of the z-differences, we would expect that w.l.o.g. $\:{\overline{x}}_{T}>{\overline{x}}_{C}$ or $\:{\widehat{p}}_{T}>{\widehat{p}}_{C}$ respectively. It follows, that $\:{z}_{con}>0$ (or $\:{z}_{bin}>0$ reps.) and consequently large initial values of $\:SS{Q}_{zDiff}$ are observed at the beginning of *Dynamic Landmarking*, that is, before the first deletion step.

When adding a correlation between $\:U$ and $\:X$, we find that the estimated treatment effects becomes closer to the true, simulated treatment effect, the stronger the correlation. In addition, the $\:SS{Q}_{zDiff}$ come closer to being balanced after PS matching as correlation increases. This is because the omitted covariate $\:U$ will be matched in parallel with the true confounder$\:\:X$, if $\:U$ and $\:X$ are correlated [e.g., 33, 37].

## Illustration of the procedure with an example from cardiac surgery

We now apply the *Dynamic Landmarking* approach to individual patient data from a non-randomized trial on aortic valve implantation in cardiac surgery [12]. Here, the effect of transcatheter (either transapical (TA) or transfemoral (TF)) aortic valve implantation (TAVI) in comparison to a conventional surgical treatment (minimally invasive aortic valve replacement (MIC-AVR)) in patients with moderate surgical risk was investigated. In the original analysis, the authors used 23 baseline covariates and a 1:1:1 PS-matching algorithm for the three treatments TA-TAVI, TF-TAVI, and MIC-AVR to evaluate treatment effects by fitting stratified Cox models to the matched data set. For our investigation here, we will concentrate on the two-group comparison of MIC-AVR vs. TA-TAVI. Comparing a catheter-based intervention versus a surgical approach is of special methodological interest, because the treatments are applied to distinctly different patient populations. Unlike surgical interventions, catheter-based aortic valve implantation does not require opening the chest (sternotomy), making it suitable for much more medically compromised patients, often referred to as “high-risk patients”. For this reason, strong confounding is to be expected. Indeed, in the original analysis we already noted that the overlap of the logit-transformed PS is very small before PS matching and covariates are heavily imbalanced between intervention groups. Additionally, a univariable Cox model with treatment as the only covariate and overall survival as outcome, showed an extremely strong effect of a hazard ratio of 6.40 (95%CI: 5.33; 7.69) for the MIC-AVR group in comparison to the TA-TAVI group. After PS matching with 13 randomly selected covariates (see Table 1 for details) the hazard ratio reduced to 2.13 (95%CI 1.31; 3.45) indicating a strong influence of confounding in the crude model. Moreover, considering all 23 covariates from the original article yielded a hazard ratio of 1.64 (95%CI: 1.23; 2.19).

Given this strong degree of confounding, we use the dataset for illustrative purposes and assess it in three different ways. First, a raw model (without any prior PS-matching or any other confounder adjustment) was fitted to the data set, which means that we omitted all 28 covariates from data analysis. Second, a partially PS-matched data set with 13 (out of 28) randomly included covariates was used for *Dynamic Landmarking*. Hence, 15 randomly selected covariates were omitted from data analysis. We assessed whether the selected covariates for PS matching have an influence on the results and therefore repeated the partially matching various times using different sets of randomly selected/omitted covariates. All scenarios showed similar results regarding the trajectories of *Dynamic Landmarking*; therefore, we present only one representative example in the paper (chosen covariates can be found in Table 1). In a third scenario, we reproduced the PS matching analysis from the original publication, including the 23 original and omitting the remaining five covariates. For all scenarios we used greedy nearest neighbour procedure with a caliper of width, equal to 0.2 of the standard deviation of the logit of the propensity score. Actually, the idea of *Dynamic Landmarking* is to measure the balance of omitted covariates; however, for a real data set it is also important to check the balance of the PS matched covariates. Therefore, we present the $\:SS{Q}_{zDiff}\:$in Section “Patients’ characteristics before and after PS matching” for both, included and omitted covariates. For better clarity, we introduce a special notation to separate included and omitted covariates for each scenario: An x/y-scenario describes a scenario were ‘x’ covariates are included in the PS model and ‘y’ covariates are omitted from the data analysis but are used for balance measuring during *Dynamic Landmarking*. Analogously, $\:SS{Q}_{zDiff}\left(x\right)/SS{Q}_{zDiff}\left(y\right)\:$describes the sum of squared z-differences for the (‘x’ included)/(‘y’ omitted) covariates. Table 1 summarizes the three scenarios.

**Table 1:** Notation for scenarios

Scenario	Description	Matched covariates	Notation scenario	Notation SSQ_z__Diff_
I	No (0) covariates are included in the PS model (raw analysis without any PS-matching); 28 covariates are omitted from data analysis	-	0/28-scenario	- /SSQ_z__Diff_(28)
II	13 covariates are included in the PS model; 15 covariates are omitted from the data analysis	Gender, weight, euroSCORE II, German Aortic valve score, STS score, Hypertension, pulmonary hypertension, Stroke, PAOD, Cerebrovascular disease, Atrial fibrillation, Previous MI, NYHA class	13/15-scenario	SSQ_z__Diff_(13) /SSQ_z__Diff_(15)
III	23 covariates are included in the PS model; 5 covariates are omitted from the data analysis	Covariates from scenario II, Age, year of surgery, height, LVEF, GFR, Previous aortic valve surgery, DM, COPD, CAD, priority urgent	23/5-scenario	SSQ_z__Diff_(23) /SSQ_z__Diff_(5)

### Patients’ characteristics before and after PS matching

Table 2 summarizes the preoperative patient characteristics for each scenario. Unsurprisingly, most of the characteristics are extremely imbalanced without PS matching (0/28-scenario), as both groups strongly differ in their baseline characteristics ($\:SS{Q}_{zDiff}$: - / 6,538.44). In the 13/15-scenario, 240 pairs could be matched based on the following covariates: gender, weight, euroSCORE II, German aortic valve score, STS score, hypertension, pulmonary hypertension, stroke, PAOD, cerebrovascular disease, atrial fibrillation, previous MI, and NYHA class. Interestingly, the 13/15-scenario improved the balance of both, the included and omitted covariates ($\:SS{Q}_{zDiff}$: 62.20 / 476.63); however, the balance of the included covariates is still unsatisfactory, as the expected value for a perfect matching would be 6.5 for 13 matched covariates [20]. In the 23/5-scenario we utilized the same covariates as in the 13/15-scenario and additionally included age, year of surgery, height, LVEF, GFR, previous aortic valve surgery, diabetes mellitus, COPD, CAD, and priority status as covariates in the PS model. This resulted in 177 pairs hardly differing in terms of preoperative covariates and their balance ($\:SS{Q}_{zDiff}$: 27.14 / 4.66). It can be seen that the variables that were not used for PS matching in the 13/15- and 23/5-scenario nevertheless show a decreasing imbalance. This is due to the anticipated association between included and omitted covariates, which results in a parallel matching also for the omitted covariates.

### *Dynamic Landmarking* for scenario I (0/28)

**Fig. 4:**
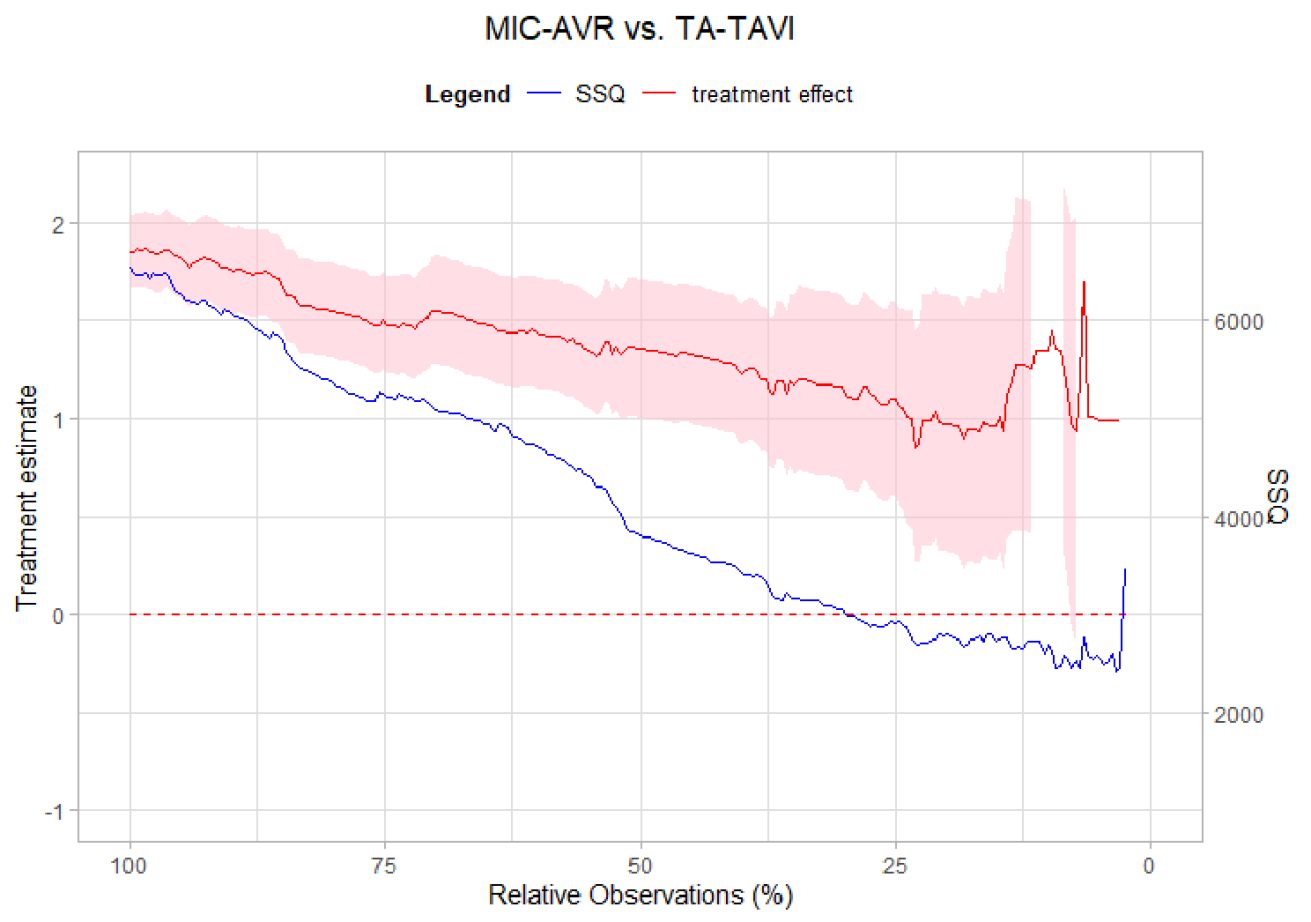
Trajectories of treatment effect (left y-axis, red) on the log(HR) scale and sum of squared z-differences (right y-axis, blue) for Scenario I (0/28)

In the first scenario, we applied *Dynamic Landmarking* for the raw model without performing any PS matching prior to fitting a univariable Cox model with treatment as the only covariate. The results can be found in Fig. 4. Not surprisingly, we observe a consistently shifting treatment effect trajectory. Upon analysing the balance of the 28 omitted covariates, we notice the very high initial values of$\:\:SS{Q}_{zDiff}$ (concrete: 6,538.44). Consequently, *Dynamic Landmarking* indicates that these omitted covariates might induce confounding bias. This results in a biased treatment effect estimate for this model (expressed as a hazard ratio of 6.40) due to confounding. One approach to rectify this bias would be to employ a PS model, taking into account the omitted covariates, before fitting the stratified Cox model.

### *Dynamic Landmarking* for Scenario II (13/15)

**Fig. 5:**
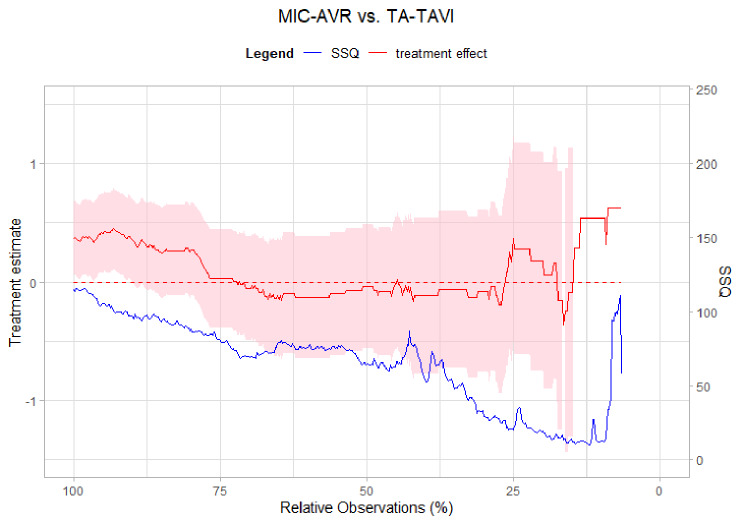
Trajectories of treatment effect (left y-axis, red) on the log(HR) scale and sum of squared z-differences (right y-axis, blue) for Scenario II (13/15)

After PS matching with 13 covariates, we applied the *Dynamic Landmarking* approach and collected the regression parameters to draw a trajectory depending on the remaining number of observations (see Fig. 5). We still observe a systematic shift in the treatment effect estimates, at least for the first 50% of deleted patients, and correspondingly a decreasing $\:SS{Q}_{zDiff\:}$during the procedure. Therefore, as expected from the simulation results, a still biased treatment effect estimate is obtained in the 13/15-scenario, pointing to confounding bias which is induced by the 15 omitted covariates. We further observe that the omitted 15 covariates also improve their balance after PS matching, indicating that included and omitted covariates are correlated. However, this correlation does not appear to be strong enough to obtain a treatment effect that is not influenced by confounding bias. Consequently, the user either needs to adjust the Cox model for the omitted confounders or must include them in the initial PS-matching. *Dynamic Landmarking* should be repeated for the enlarged confounder set to check whether the treatment effect estimate is still influenced by confounding or built-in selection bias.

### *Dynamic Landmarking* for scenario III (23/5)

**Fig. 6:**
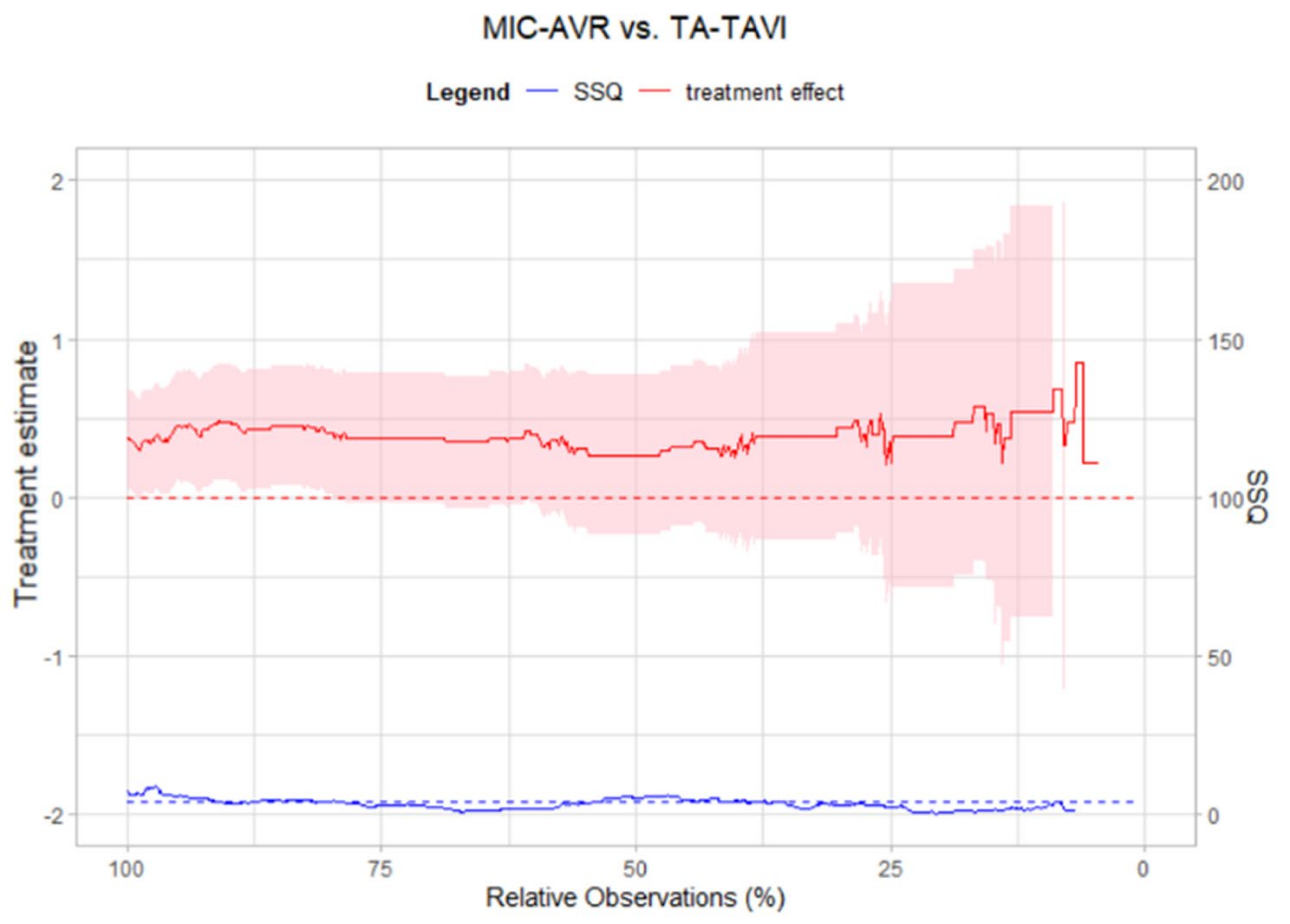
Trajectories of treatment effect (left y-axis, red) on the log(HR) scale and sum of squared z-differences (right y-axis, blue) for Scenario III (23/5)

In the last scenario, all original 23 confounders were included as covariates in the PS model. *Dynamic Landmarking* shows a treatment effect trajectory with only random fluctuations and no systematic change in the $\:SS{Q}_{zDiff}$-trajectory (see Fig. 6) in this data set. For balance fitting we used five additional covariates (MELD-Score, diameter of aortic valve, drainage quantity, haemoglobin and, creatinine level) which were measured during the trial, but not included in the original analysis by Furukawa (2018). We observe balanced covariates during the whole *Dynamic Landmarking* process, which indicates that these five covariates do not have a relevant impact on the treatment effect estimate. To summarize, we would conclude that the estimated treatment effect in the 23/5-scenario might not be subject to confounding or built-in selection bias, as no systematic shift in the treatment effect estimate can be observed.

**Table 2:** Patients’ characteristics (italic numbers are matched characteristic in each scenario)

	0/28-model (*N* = 2536)				13/15-model (*N* = 480)				23/5-model (*N* = 354)		
Variable	MIC-AVR (*n* = 1929)	TA-TAVI (*n* = 607)	z-Diff/SMD		MIC-AVR (*n* = 240)	TA-TAVI (*n* = 240)	z-Diff/SMD		MIC-AVR (*n* = 177)	TA-TAVI (*n* = 177)	z-Diff/SMD
Female	836 (43.3%)	328 (54.0%)	-4.62/-0.21		*133 (55.4%)*	*118 (49.2%)*	*1.37/0.13*		*88 (49.7%)*	*87 (49.2%)*	*-0.11/-0.01*
Weight	81.04 (± 16.12)	73.66 (± 16.06)	-9.86/-0.45		*76.17 (± 16.11)*	*76.68 (± 15.58)*	*0.35/0.03*		*76.47 (± 15.89)*	*77.17 (± 14.86)*	*-0.43/-0.04*
euroSCORE II	1.62 (± 1.44)	8.77 (± 8.87)	19.78/1.13		*3.87 (± 2.70)*	*6.80 (± 11.62)*	*3.80/0.33*		*5.42 (± 9.5)*	*3.58 (± 2.69)*	*2.48/0.21*
German Aortic Valve score	1.32 (± 0.73)	3.81 (± 3.38)	18.02/1.02		*2.35 (± 1.13)*	*3.40 (± 4.53)*	*3.48/0.31*		*3.26 (± 4.71)*	*2.32 (± 1.19)*	*2.59/0.28*
STS score	1.84 (± 1.37)	7.56 (± 5.89)	23.73/1.34		*4.01 (± 2.17)*	*5.81 (± 7.25)*	*3.69/0.31*		*5.49 (± 7.46)*	*3.97 (± 2.41)*	*2.58/0.25*
Hypertension	1447 (75.0%)	549 (90.4%)	-9.90/-0.42		*217 (90.4%)*	*213 (88.8%)*	*1.15/0.06*		*156 (88.1%)*	*157 (88.7%)*	*0.17/0.02*
Pulmonary hypertension	177 (9.2%)	202 (33.3%)	-11.9/-0.61		*56 (23.3%)*	*56 (25.3%)*	*0.00/0.00*		*42 (23.7%)*	*42 (23.7%)*	*0.00/0.00*
Stroke	37 (1.9%)	51 (8.4%)	-5.55/-0.30		*18 (7.5%)*	*23 (9.6%)*	*-1.56/-0.03*		*9 (5.1%)*	*11 (6.2%)*	*0.46/-0.04*
PAOD	60 (3.1%)	193 (31.8%)	-14.85/-0.81		*38 (15.8%)*	*40 (16.7%)*	*-0.48/-0.02*		*30 (16.9%)*	*26 (14.7%)*	*-0.58/-0.05*
Cerebrovascular disease	89 (4.6%)	140 (23.1%)	-10.39/-0.55		*36 (15.0%)*	*27 (11.3%)*	*2.47/0.09*		*22 (12.4%)*	*30 (16.9%)*	*1.20/0.11*
Atrial fibrillation	36 (1.9%)	167 (27.5%)	-13.95/-0.78		*26 (10.8%)*	*33 (13.8%)*	*-1.86/0.07*		*22 (12.4%)*	*20 (11.3%)*	*-0.33/0.03*
Previous MI	58 (3.0%)	100 (16.5%)	-8.66/-0.46		*12 (5.0%)*	*14 (5.8%)*	*-0.78/-0.06*		*15 (8.5%)*	*15 (8.5%)*	*0.00/0.00*
NYHA class I II III IV	219 (11.3%) 983 (51.0%) 700 (36.3%) 27 (1.4%)	20 (3.3%) 174 (28.7%) 345 (56.8%) 68 (11.2%)	-14.34/0.47		*6 (2.5%)* *97 (40.4%)* *119 (49.6%)* *18 (7.5%)*	*12 (5.0%)* *79 (32.9%)* *131 (54.6%)* *18 (7.5%)*	*-0.72/0.04*		*12 (6.8%)* *58 (32.8%)* *97 (54.8%)* *10 (5.6%)*	*6 (3.4%)* *73 (41.2%)* *86 (48.6%)* *12 (6.8%)*	*-0.44/0.01*
Age	67.85 (± 10.98)	81.28 (± 6.08)	38.24/1.51		76.78 (± 6.42)	80.59 (± 6.07)	6.68/0.61		*79.38 (± 6.46)*	*78.29 (± 5.53)*	*1.71/0.18*
Year of surgery 2009 2010 2011 2012 2013 2014 2015 2016 2017	74 (3.8%) 146 (7.6%) 168 (8.7%) 218 (11.3%) 273 (14.2%) 352 (18.3%) 323 (16.7%) 236 (12.2%) 139 (7.2%)	16 (2.6%) 41 (6.8%) 49 (8.1%) 76 (12.5%) 97 (16.0%) 113 (18.6%) 121 (19.9%) 53 (8.7%) 41 (6.8%)	0.10/0.03		4 (1.6%) 22 (9.2%) 23 (9.6%) 27 (11.3%) 29 (12.1%) 51 (21.3%) 38 (15.8%) 31 (12.9%) 15 (6.3%)	9 (3.8%) 11 (4.6%) 20 (8.3%) 28 (11.7%) 42 (17.5%) 48 (20.0%) 56 (23.3%) 12 (5.0%) 14 (5.8%)	0.32/0.18		*7 (4.0%)* *7 (4.0%)* *15 (8.5%)* *24 (13.6%)* *30 (16.9%)* *29(16.4%)* *43 (24.3%)* *12 (6.8%)* *10 (5.6%)*	*7 (4.0%)* *18 (10.2%)* *14 (7.9%)* *19 (10.7%)* *27 (15.3%)* *37 (20.9%)* *29 (16.4%)* *17 (9.6%)* *9 (5.1%)*	*-0.81/0.09*
Height	170.53 (± 9.51)	165.49 (± 9.45)	-11.45/-0.53		166.75 (± 9.43)	167.29 (± 9.67)	0.61/0.06		*166.98 (± 10.07)*	*167.47 (± 8.96)*	*-0.49/-0.05*
LVEF	60.94 (± 9.29)	51.25 (± 12.16)	-18.03/-089		58.01 (± 10.23)	53.83 (± 11.42)	-4.22/-0.39		*55.95 (± 9.93)*	*56.15 (± 10.78)*	*-0.18/-0.02*
GFR	78.74 (± 20.25)	55.83 (± 22.81)	-22.12/-1.06		60.45 (± 23.16)	64.64 (± 20.81)	2.09/0.19		*63.78 (± 22.63)*	*64.77 (± 23.43)*	*-0.41/-0.04*
Previous aortic valve surgery	1 (0.1%)	13 (2.1%)	-3.54/-0.20		1 (0.4%)	3 (1.3%)	-1.76/-0.09		*1 (0.5%)*	*1 (0.5%)*	*0.00/0.00*
Diabetes mellitus	362 (18.8%)	214 (35.3%)	-7.73/-0.38		72 (30.0%)	61 (25.4%)	2.23/0.10		*53 (29.9%)*	*50 (28.2%)*	*-0.35/-0.04*
COPD	88 (4.6%)	105 (17.3%)	-7.93/-0.47		34 (14.2%)	27 (11.3%)	1.93/0.09		*21 (11.8%)*	*21 (11.8%)*	*0.00/0.00*
CAD 1-vessel 2-vessel 3-vessel	171 (8.9%) 75 (3.9%) 46 (2.4%)	99 (16.3%) 83 (13.7%) 214 (35.3%)	-25.94/-0.22		37 (15.4%) 17 (7.1%) 13 (5.4%)	46 (19.2%) 29 (12.1%) 57 (23.8%)	-6.66/-0.10		*27 (15.3%)* *20 (11.3%)* *32 (18.1%)*	*32 (18.1%)* *20 (11.3%)* *24 (13.6%)*	*-0.67/-0.08*
Priority urgent (emergency)	9 (0.5%)	14 (2.3%)	-2.93/-0.16		3 (1.3%)	8 (3.3%)	-2.70/-0.14		*5 (2.8%)*	*3 (1.7%)*	*-0.72/-0.08*
MELD-Score	7.54 (± 2.16)	8.27 (± 4.98)	-3.51/-0.27		9.41 (± 3.77)	9.75 (± 4.53)	-0.87/-0.16		9.69 (± 4.36)	9.05 (± 3.37)	1.50/0.23
Diameter of aortic valve	23.47 (± 1.89)	25.88 (± 2.07)	-25.52/-1.72		22.79 (± 1.81)	26.02 (± 2.06)	-18.24/-2.36		26.04 (± 2.07)	25.89 (± 1.83)	0.06/0.11
Drainage quantity	420.83 (± 328.31)	486.38 (± 429.62)	-2.56/-0.24		458.30 (± 391.12)	489.45 (± 415.36)	-0.84/-0.11		462.20 (± 357.03)	471.13 (± 366.32)	-0.23/-0.03
preoperative haemoglobin level	13.77 (± 1.51)	12.26 (± 1.69)	7.27/1.33		12.80 (± 1.77)	12.46 (± 1.78)	2.06/0.27		12.5 (± 1.72)	12.77 (± 1.71)	-1.47/-0.22
preoperative creatinine level	0.99 (± 0.49)	1.45 (± 1.08)	-10.92/-0.78		1.34 (± 0.99)	1.14 (± 0.47)	2.74/0.36		1.24 (± 0.85)	1.20 (± 0.85)	0.44/0.07

## Discussion

*Dynamic Landmarking* can be used in PS matched analysis as a post-hoc diagnosing tool to visualize if the estimated treatment effects from a Cox model thread to confounding or built-in selection bias. Furthermore, the approach can give a hint on whether prognostic factors or confounders have been omitted from the data analysis. Depending on the causal direction of the omitted covariate, different issues could arise. While an omitted prognostic factor would induce built-in selection bias, resulting in a difference between conditional and marginal treatment effect, the omssion of confounders would result in confounding bias. We showed by simulation that *Dynamic Landmarking* indeed is able to visualize and distiguish between both issues, at least in case of independent omitted covariates. More precisely, both built-in selesction bias and confounding bias show systematically changing treatment effect trajectories during *Dynamic Landmarking*. Furthermore, omitted confounders tend to be heavily unbalanced between the groups yielding high initial $\:SS{Q}_{zDiff}$- values for the full PS matched data set. On the other hand, prognostic are still balanced after PS-matching, yielding small $\:SS{Q}_{zDiff}$-values at the Beginning of *Dynamic Landmarking*, but showing an increasing imbalance for the first 50% of deleted observations while the procedure continues. This is what previous work also showed for RCTs [32]. Please note that, while an inspection of the initial $\:SS{Q}_{zDiff}$-values give a first hint on the causal direction of the omitted covariate, it is important to consider both. This is because omitted instrumental variables (i.e., $\:{\beta\:}_{U}=0,\:{\alpha\:}_{U}\ne\:0$) would show high intial $\:SS{Q}_{zDiff}$-values. However, in such cases the treatment effect trajectory will remain stable with only random fluctuations (see supplement, Fig. S11).

**Fig. 7:**
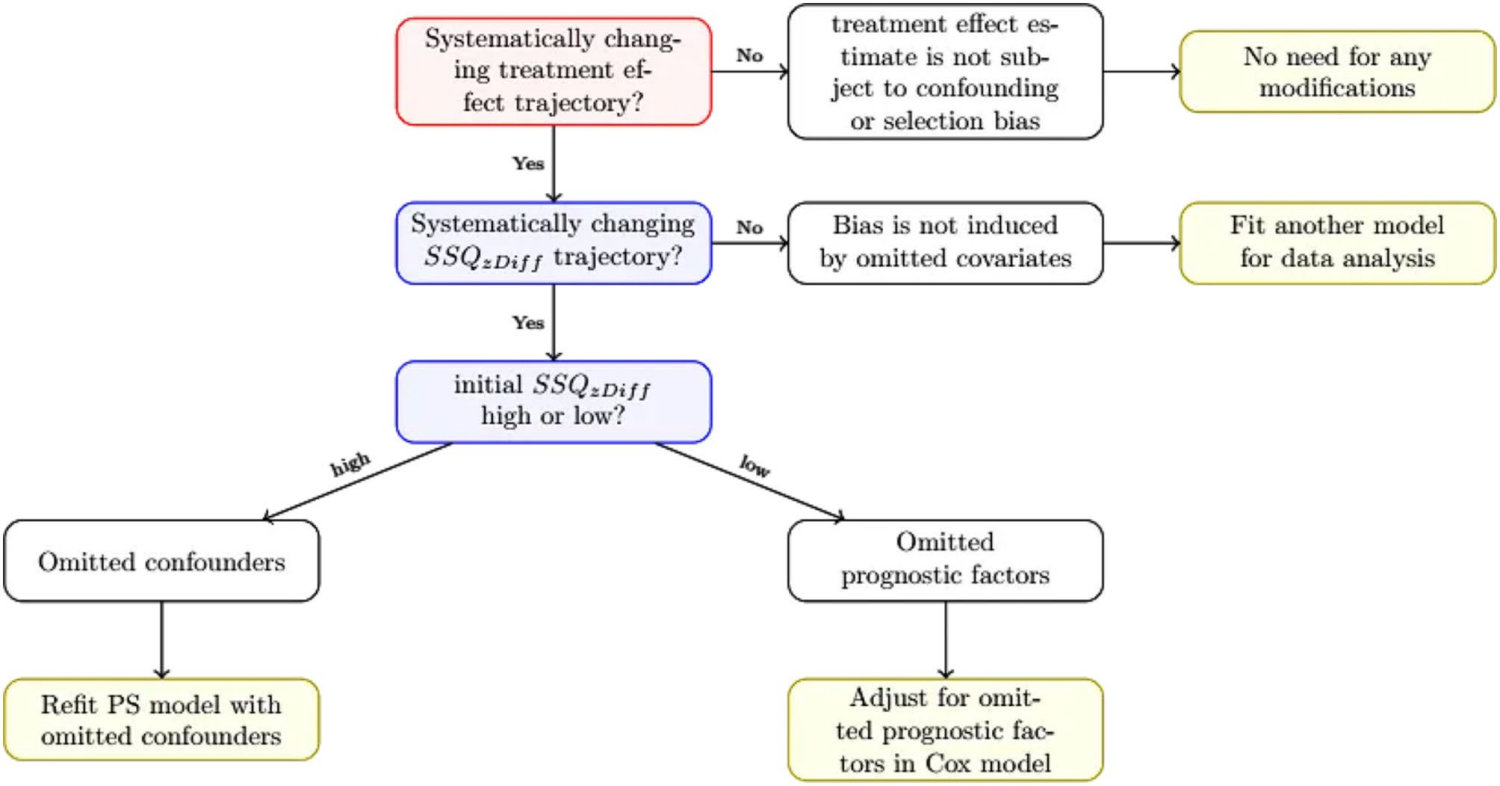
Interpretation and recommendation for Dynamic Landmarking results under the assumption of uncorrelated omitted covariates. Red boxes are related to treatment effect trajectories, blue boxes are related to $\:SS{Q}_{zDiff}$-trajectories. Grey boxes give possible interpretations for course of trajectories and green boxes are recommendations for data analysis

For omitted covariates, that were independent from included ones, we provide an interpretation- and decision-scheme for *Dynamic Landmarking* (see Fig. 7). We suggest to analyse the visual output of *Dynamic Landmarking* in a two-step-algorithm: First the treatment effect trajectory has to be regarded. Only if a systematic shift is observed in the treatment effect trajectory the $\:SS{Q}_{zDiff}$-trajectory should be involved and interpreted as mentioned. Moreover, to differentiate correctly between built-in selection and confounding bias, the user has to run the Dynamic Landmarking with each omitted covariate seperatly. Please note, that it might be possible to observe a systematically changing treatment effect tajectory, but no change in the $\:SS{Q}_{zDiff}$-trajectory. In such cases we would conclude, that the treatment effect still cannot be interpreted as time-invariant effect, but it is not possible to identify omitted covariates causing this (e.g., there might be some true unobserved/unmeasured confounders or prognostic factors [17, 36] which have to be accounted for).

In case the omitted covariate(s) are correlated with one or more considered confounders from the PS model, confounder bias or built-in selection bias can be minimized [11, 14, 15]. Rubin and Thomas (1996) stated that ”excluding potentially relevant variables should be done only [.] when the excluded variables are highly correlated with variables already in the propensity score model” [27]. Indeed, recent work found that replacing a highly correlated (namely, 0.8) covariate instead of the true confounder in the PS model would result in a relative bias less than 5% [14]. Due to the correlation, the omitted covariate will indirectly accounted for in the PS model. This result is reflected in the observed behaviour of the $\:SS{Q}_{zDiff}\:$trajectories: The stronger the correlation between matched confounder and omitted covariate, the more balanced is the omitted covariate – at least at the initial state of the *Dynamic Landmarking* procedure.

The primary focus of *Dynamic Landmarking* is on assessing the estimated treatment effect, which is why the treatment effect trajectories should be examined first when using this approach. Additionally, it can provide insights into omitted covariates that might need to be included in the analysis. However, the approach should not be compared or equated with variable selection methods. While variable selection aims to identify an appropriate set of covariates before data analysis [e.g. 13, 16] *Dynamic Landmarking* serves as a post-hoc tool to verify whether the model assumptions and corresponding effect estimates are valid. We believe that our approach should be viewed as a complement to, rather than a replacement for, such analyses.

By our empirical example we showed how induced confounder bias impacted both, treatment effect and $\:SS{Q}_{zDiff}$-values. Indeed, the omission of true confounders led to a systematically changing treatment effect trajectory and a high intial $\:SS{Q}_{zDiff}$- values. Additionally, it is important to note that although the omitted confounders are correlated with the matched confounders, this correlation alone is insufficient for obtaining an estimate of the treatment effect that is not subject to confounding bias, as showed in Fig. 5. In practice, one should estimate the PS again, including the omitted confounders in the PS model and check by a repeated run of *Dynamic Landmarking*, whether the estimates are still biased (results see Fig. 6). Of course, in real life the user would not intentionally induce bias by omitting confounders, but would immediately assess a well-specified PS model using *Dynamic Landmarking*. If no constant treatment effect trajectory can be obtained by our approach we would conclude, that other assumptions (e.g., real unobserved covariates or a time-dependent treatment effect) might be an explanation for the systematic shift. In that case, a more flexible model, e.g., time-dependent propensity score [35] or frailty modelling [36], may be used for data analysis.

We have to acknowledge some limitations of our work. First, *Dynamic landmarking* is based on the assumption that the conditional treatment (conditional on all relevant prognotic factors) is constant over time, implying proportional hazards in the data. If this is true, then the method is a good diagnostic tool for identifying whether a treatment estimate from the Cox model underlies confounding or built-in selection bias. In practice, however, time-dependent treatment effects may be observed. It is already known that it is not possible to distinguish between time-dependent treatment estimates (i.e. non-proportional hazards) and induced heterogeneity (built-in selection bias) [4, 10, 24]. In fact, this is also true for our method. Therefore, as with other methods, an assumption about the true effect (here, being constant over time and across the population) has to be made.

Second, the $\:SS{Q}_{zDiff}$ is an aggregated balance measure summarizing the global balance of all omitted covariates. We showed that the intial $\:SS{Q}_{zDiff}$ can be used to distinguish between built-in selection bias and confounding bias. We analyzed these two issue by separate simulation scenarios. In pratice, however, both issue can occur at the same time and consequently the $\:SS{Q}_{zDiff}$ may be estimated for prognostic factors as welll as confounders and summarized in one number. It should then be noted that the z-difference of confounders dominates the value of the $\:SS{Q}_{zDiff}$, as it is naturally larger than the z-difference of a prognostic factor. This can complicate the interpretation of the approach in such scenarios. One way to correctly distinguish the two effects would be to separately perform *Dynamic Landmarking* for each omitted covariate.

Third, we focused here on a specific PS method (PS-matching). Generally, PS-matching has some limitations per se, which have been discussed previously in literature [18, 34] and could also be present in our work. Related to that, we believe that recent results for optimal and matching weights will lead to increasing use of PS-weighting techniques at the cost of PS-matching [22, 23]. It seems of further interest to investigate how *Dynamic Landmarking* will perform in such situations.

## Conclusion

Overall and to summarize, we feel that *Dynamic Landmarking* is a good visual tool to verify if a Cox model used provides a treatment estimate that is not subject to confounding or built-in selection bias in PS matched trials. One substantial assumption for a valid interpretation of the resulting hazard ratio is that all relevant confounders are considered and no prognostic factors is omitted. In practice, however, it will hardly be possible to efficiently collect all covariates, confounders as well as prognostic factors. While the literature suggests that PS-matching can yield valid results in the presence of omitted variables if they are correlated with the matched confounders, this assertion is applicable only in cases of exceptionally strong correlations, which are uncommon in practical scenarios [20]. Furthermore, data collection often involves gathering more variables than those used in the final analysis. The choice of covariates for PS matching and subsequent analysis relies on current scientific understanding and clinical expertise, but it is also influenced by the researcher. Consequently, there is a possibility that omitted covariates, which were measured but not considered, may introduce built-in selection bias or confounding bias. This is precisely where *Dynamic Landmarking* comes into play, providing an opportunity to examine whether (and if so, which) covariates could distort the treatment effect estimate.

## Electronic Supplementary Material

Below is the link to the electronic supplementary material.

Supplementary Material 1

## Data Availability

The datasets used and/or analysed during the current study are available from the corresponding author on reasonable request.

## Abbreviations

RCT: Randomized controlled trials
PS: Propensity score
SSQ_z__Diff_: Sum of squared z-difference
TA: Transapical
TF: Transfemoral
TAVI: Transcatheter aortic valve implantation
MIC-AVR: Minimally invasive aortic valve replacement
