# Supplementary material for: Built-in selection or confounder bias? Dynamic Landmarking in matched propensity score analyses
Source: BMC Med Res Methodol. 2024 Dec 21;24:316. doi: 10.1186/s12874-024-02444-7 (PMC11662801; doi:10.1186/s12874-024-02444-7)
Supplement: Supplementary file 1 — Supplementary Material 1 [file 12874_2024_2444_MOESM1_ESM.docx]

**Supplement**

**Tab S1: Definition of terminology used in the manuscript**

| **Term** | **Definition** |  |
| --- | --- | --- |
| **Prognostic factor** | A ***prognostic factor*** $U$is a variable associated with a subsequent time-to-event outcome$Y$, but not associated with treatment allocation$Z$. | 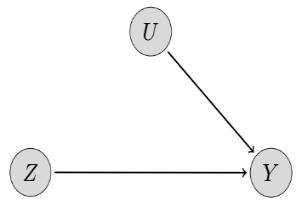 |
| **Confounder** | A ***confounder*** $U$is a variable associated with the treat-ment allocation$Z$ and a subsequent time-to-event outcome$Y$. | 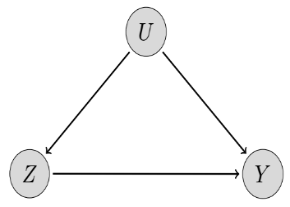 |
| **Instrumental variable** | A ***instrumental variable*** $U$ is a variable associated with the treatment allocation $Z$, but not associated with the time-to-event outcome $Y$ | 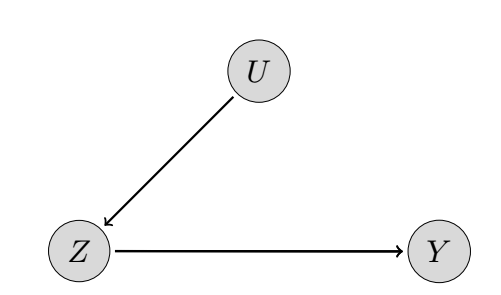 |
| **Covariate** | The term ***covariate*** is used if statements made include more than one category of the abovementioned (i.e. a covariate can be a prognostic factor, a confounder or a instrumental variable and therefore the term does not determine the causal direction) |  |
| **Conditional hazard** | Consider a proportional hazards model for two covariates: A treatment $Z$ and a prognostic factor $U$ with corresponding regression coefficients $\beta_{Z} \mathrm{and} \beta_{U}$. The ***conditional hazard*** is defined as  $\lambda\left( t \vert Z, U \right)= \lambda_{0}\left( t \right)\exp(\beta_{Z}Z+ \beta_{U}U)$  with $\beta_{Z}$ summarizing the conditional treatment effect of $Z$ (conditional with respect to $U$). The conditional treatment effect can be estimated via adjusted Cox regression (including all relevant prognostic factor). It summarises the effect on subject-specific level, i.e., what effect can be expected when moving an individual from treated to untreated. | 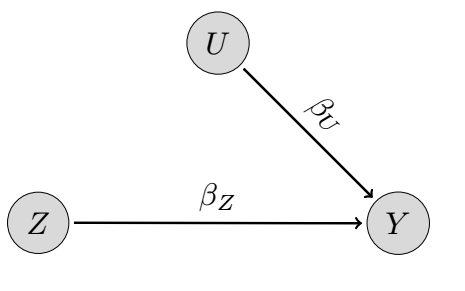 |
| **Marginal hazard** | Consider a proportional hazards model for two covariates: A treatment $Z$ and a prognostic factor $U$ with corresponding regression coefficients $\beta_{Z} \mathrm{and} \beta_{U}$. The ***marginal hazard*** is defined as  $\lambda\left( t \vert Z \right)= \lambda_{0}\left( t \right)\exp(\beta_{Z}Z)$  It summarises the effect on average-population level, i.e., what effect can be expected when moving a whole population from treated to untreated. | 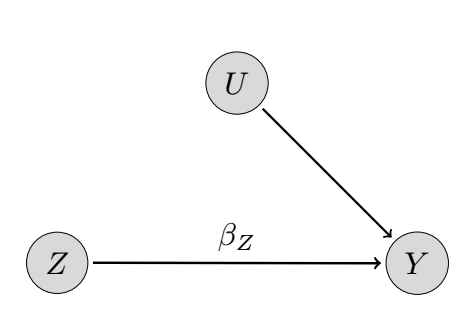 |
| **Non-collapsibility** | ***Non-collapsibility*** referes to the circumstance where conditional and marginal treatment effects are not the same when control is made for a relevant prognostic factor $U$ in the conditional model. Please note, in the special case that no pognostic factor $U$ exists, the marginal and conditional model would be the same and marginal and conditional treatment effect are the same. |  |
| **Built-in selection bias** | Assume the exstence of a prognostic factor $U$. The omission of $U$ in the Cox model would induce heterogeneity in baseline risk regarding $U$ between patients. This results in ***built-in selection bias*** as the hazard ratio is estimated conditioning on past survival. |  |
| **Propensity Score** | ***Propensity Score (PS)*** defines the individual-specific probability of receiving a treatment conditional on a set of baseline characteristics $X_{i}$:  $P\left( Z=1 \right\vert X_{i}).$  Four PS methods are widely used in medical research: matching, stratfication, adjustment and IPTW. All methods assume positivity, consistency and unconfoundedness (e.g., all relevant confounders have to measured and included in the PS model). |  |
| **Confounding bias** | Assume a non-randomized trial with binary treatment $Z$ and two confounders $X$ and $U$. The true PS would be $PS_{1}=P\left( Z=1 \right\vert X, U)$.  Assume w.l.o.g. the omission of confounder $U$ from the PS model, i.e., $PS_{2}= P(Z=1\vert X)$.  Matching on $PS_{1}$ or $PS_{2}$and estimating a Cox model which only includes the treatment $Z$ would result in different treatment estimates. The magnitude of this contrast is referred to as ***confounding bias*** *(Note: we assume here the absence of prognotic factors).* | 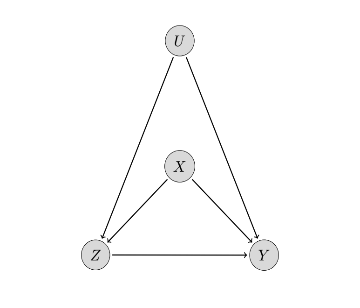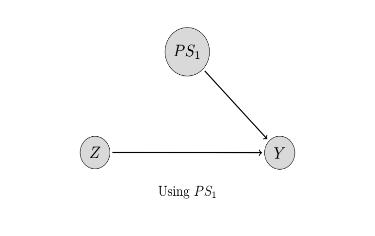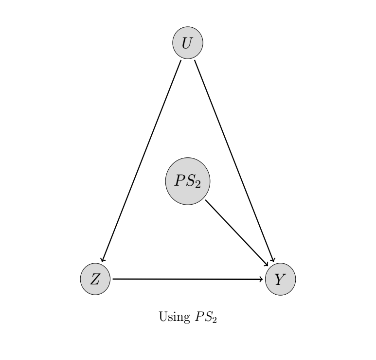 |

**Notes:**

1. Built-in selection bias can arise without confounding bias and confounding bias can arise without built-in selection bias. In RCTs, by design, only built-in selection bias can occur. In PS matched trials, however, both issues (or only one of them or even none of them) can occur.
2. Typically, in RCTs and PS-matched trials, a marginal Cox model without any adjustments is fitted. However, when aiming for a conditional treatment effect in both study designs, all relevant prognostic factors (if present) must be taken into account by including them in the Cox model

**Tab S2: Distinction of terminology and concepts**

|  | **RCT** | | **Matched PS Analysis** | |
| --- | --- | --- | --- | --- |
| Threat from confounding bias | No | | Yes, if confounders are omitted | |
| Adjustment for confounders | Not needed | | Via the PS model | |
| Propensity Score | Fixed and known (0.5 in a 1:1-randomized trial) | | Estimated from the data via logistic regression | |
| **Estimate** | **Marginal** | **Conditional** | **Marginal** | **Conditional** |
| Threat from selection bias/non-collapsibility | Yes, if prognostic factors are omitted | | | |
| Classical interpretation | Population-averaged | Subject-specific | Population-averaged | Subject-specific |
| Covariates in the Cox model | Treatment only^[[1]](#footnote-1)^ | Treatment plus all relevant prognostic factors | Treatment only^1^ | Treatment plus all relevant prognostic factors |
| Adjustment for prognostic factors | No^1^ | Yes | No^1^ | Yes |

**Formula S1: Calculation of z-differences for all scales**


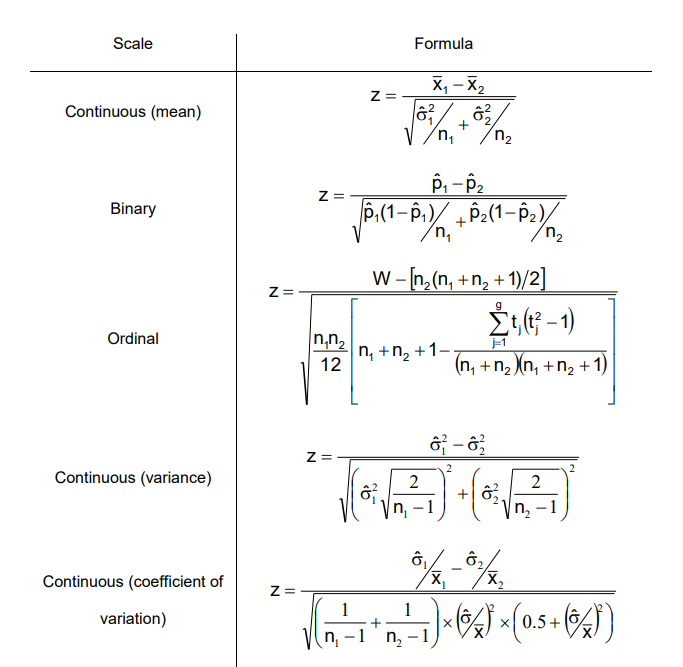


Here $\bar{x}_{1}, \bar{x}_{2}, \hat{\sigma}_{1}^{2}, \hat{\sigma}_{2}^{2}, \hat{p}_{1}, \hat{p}_{2}, N_{1}, N_{2}$denote the respective estimated means, variances, proportions, and sample sizes of the two groups. $W$ equals the sum of ranks in group 2, where ranks have been taken with values from both groups amalgamated, $g$ is the number of different values in the data set and $t_{j}$ is the number of identical values $j\left( 24 \right)$.


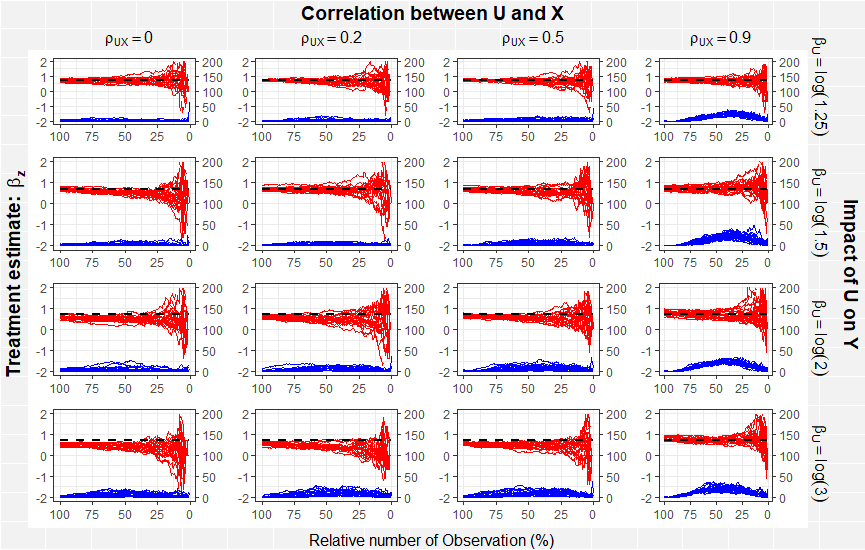


**Fig. S1:** Trajectories of treatment effect (left y-axis, red) on the log(HR) scale and sum of squared z-differences (right y-axis, blue) for balance measuring of the omitted covariate $U$ for the 500 simulated data sets. Dashed black lines show the true, conditional treatment effect estimate$\beta_{Z}=\log\left( 2 \right)$. All scenarios assume the omission of a prognostic factor $U$, i.e. $\alpha_{U}=0$., and a censoring rate of 10%.


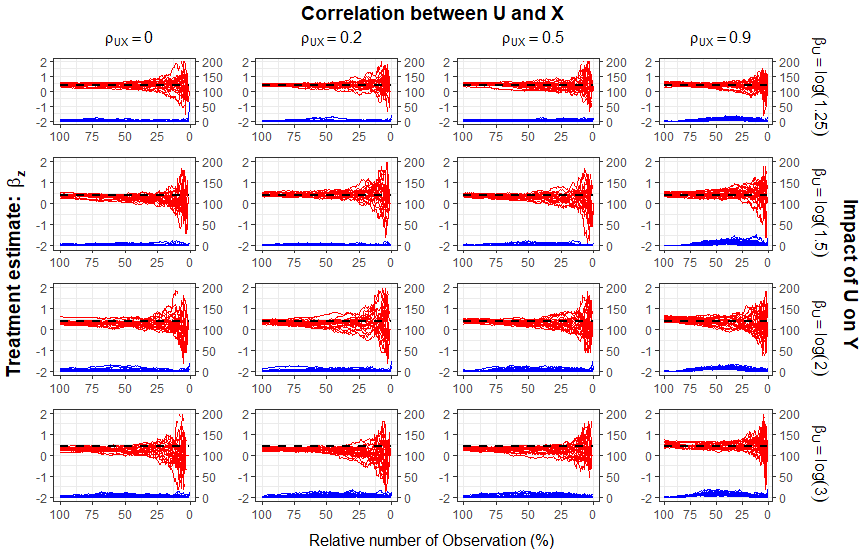


**Fig. S2:** Trajectories of treatment effect (left y-axis, red) on the log(HR) scale and sum of squared z-differences (right y-axis, blue) for balance measuring of the omitted covariate $U$ for the 500 simulated data sets. Dashed black lines show the true, conditional treatment effect estimate$\beta_{Z}=\log\left( 1.5 \right)$. All scenarios assume the omission of a prognostic factor $U$, i.e. $\alpha_{U}=0$., and a censoring rate of 10%.


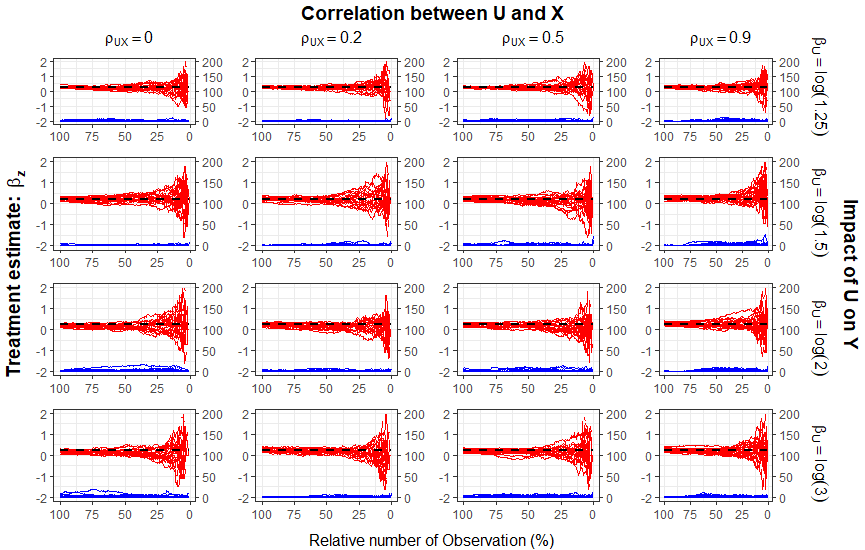


**Fig. S3:** Trajectories of treatment effect (left y-axis, red) on the log(HR) scale and sum of squared z-differences (right y-axis, blue) for balance measuring of the omitted covariate $U$ for the 500 simulated data sets. Dashed black lines show the true, conditional treatment effect estimate$\beta_{Z}=\log\left( 1.25 \right)$. All scenarios assume the omission of a prognostic factor $U$, i.e. $\alpha_{U}=0$., and a censoring rate of 10%.


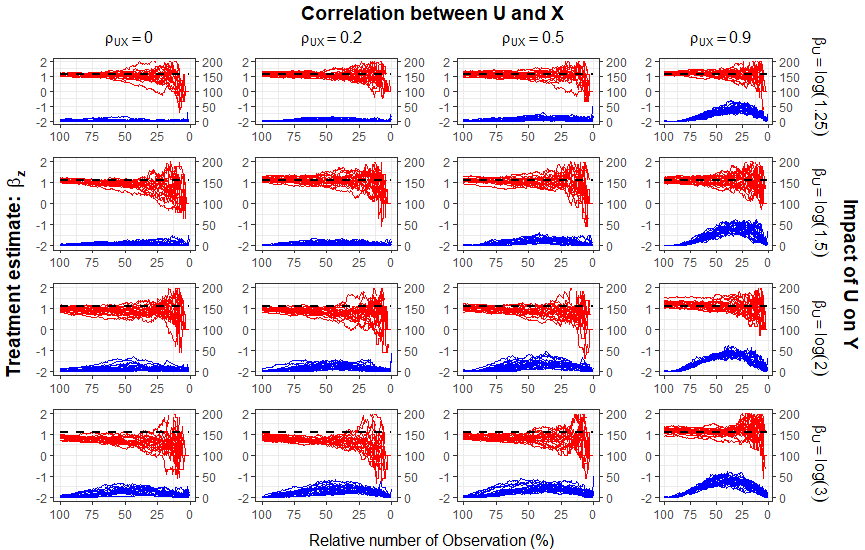


**Fig. S4:** Trajectories of treatment effect (left y-axis, red) on the log(HR) scale and sum of squared z-differences (right y-axis, blue) for balance measuring of the omitted covariate $U$ for the 500 simulated data sets. Dashed black lines show the true, conditional treatment effect estimate$\beta_{Z}=\log\left( 3 \right)$. All scenarios assume the omission of a prognostic factor $U$, i.e. $\alpha_{U}=0$., and a censoring rate of 40%.


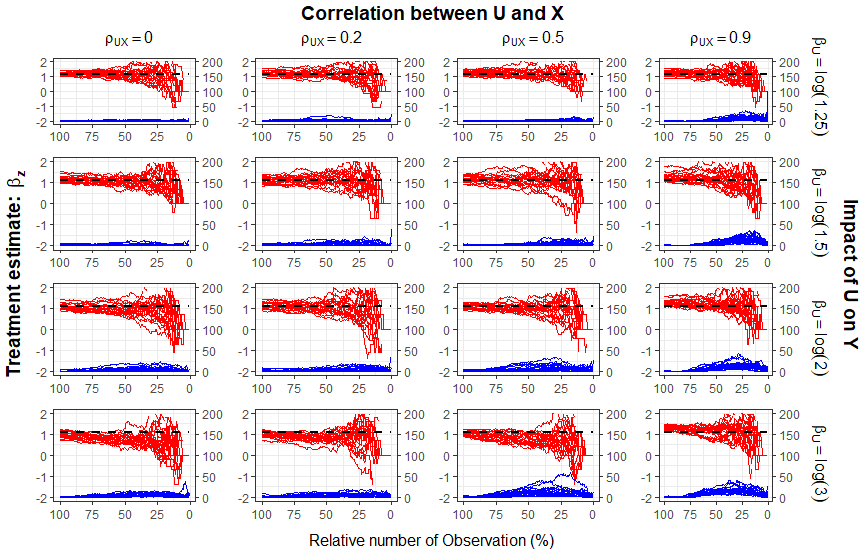


**Fig. S5:** Trajectories of treatment effect (left y-axis, red) on the log(HR) scale and sum of squared z-differences (right y-axis, blue) for balance measuring of the omitted covariate $U$ for the 500 simulated data sets. Dashed black lines show the true, conditional treatment effect estimate$\beta_{Z}=\log\left( 3 \right)$. All scenarios assume the omission of a prognostic factor $U$, i.e. $\alpha_{U}=0$., and a censoring rate of 80%.


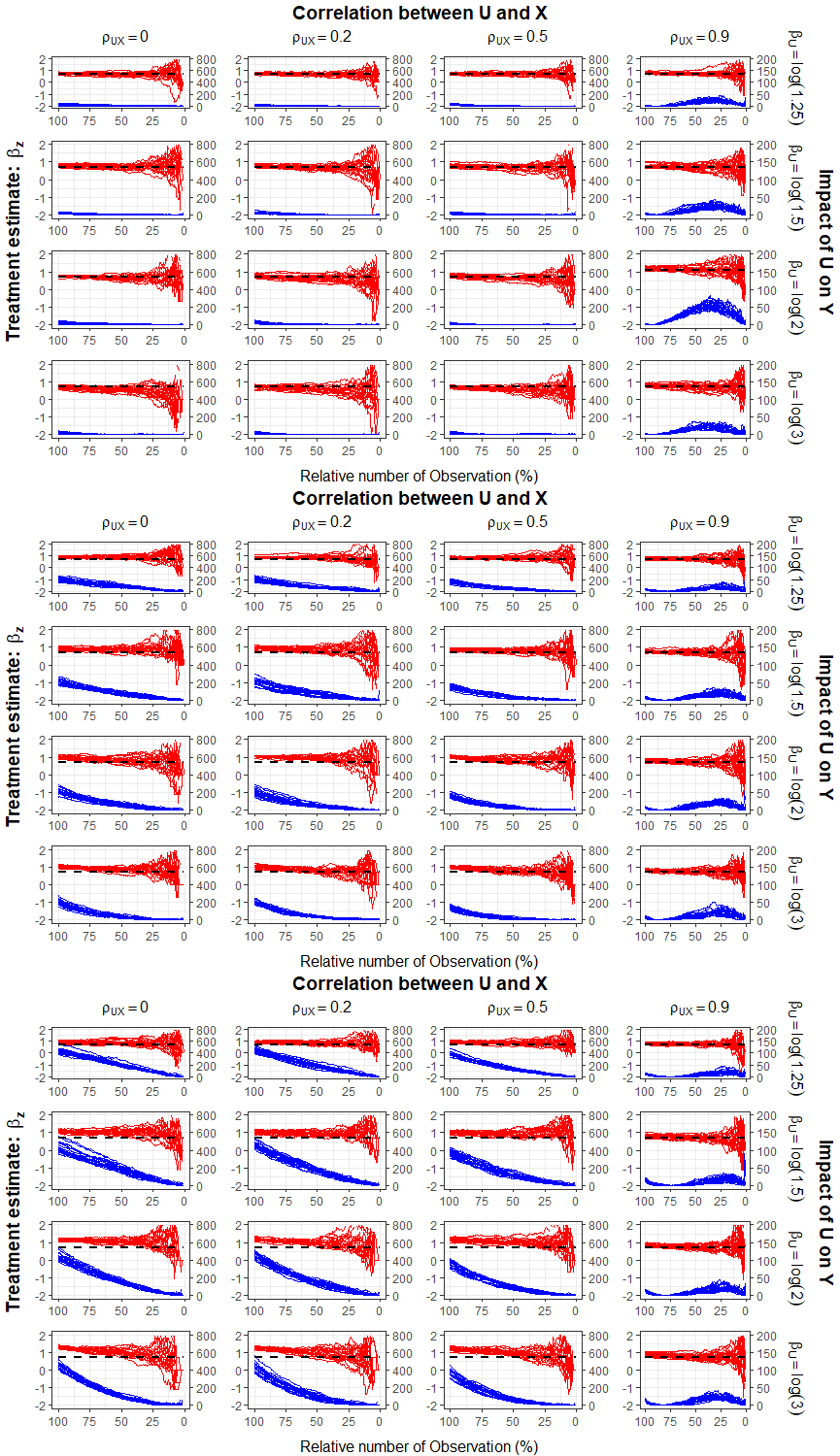


**Fig. S6:** Trajectories of treatment effect (left y-axis, red) on the log(HR) scale and sum of squared z-differences (right y-axis, blue) for balance measuring of the omitted covariate $U$ for the 500 simulated data sets. Dashed black lines show the true, conditional treatment estimate$\beta_{z}= log \left( 2 \right)$. All scenarios assume the omission of a true confounder $U$ with **A**: low impact on treatment allocation, i.e., $\alpha_{u}=\log\left( 1.25 \right)$ **B**: moderate impact on treatment allocation, i.e., $\alpha_{U}=\log\left( 2 \right)$. **C**: high impact on treatment allocation, i.e. $\alpha_{U}=\log\left( 3 \right)$


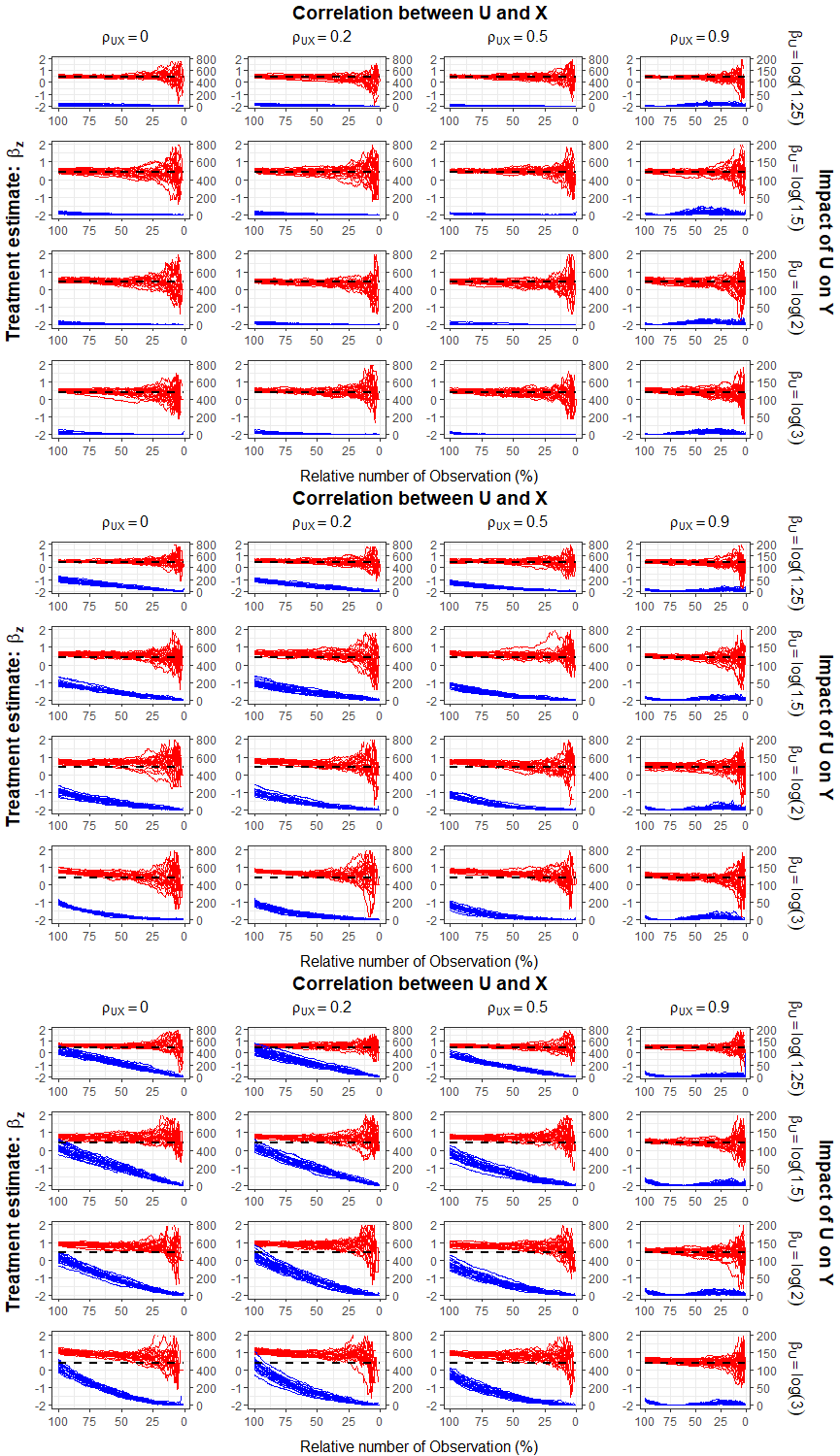


**Fig. S7:** Trajectories of treatment effect (left y-axis, red) on the log(HR) scale and sum of squared z-differences (right y-axis, blue) for balance measuring of the omitted covariate $U$ for the 500 simulated data sets. Dashed black lines show the true, conditional treatment estimate$\beta_{z}= log \left( 1.5 \right)$. All scenarios assume the omission of a true confounder $U$ with **A**: low impact on treatment allocation, i.e., $\alpha_{u}=\log\left( 1.25 \right)$ **B**: moderate impact on treatment allocation, i.e., $\alpha_{U}=\log\left( 2 \right)$. **C**: high impact on treatment allocation, i.e. $\alpha_{U}=\log\left( 3 \right)$


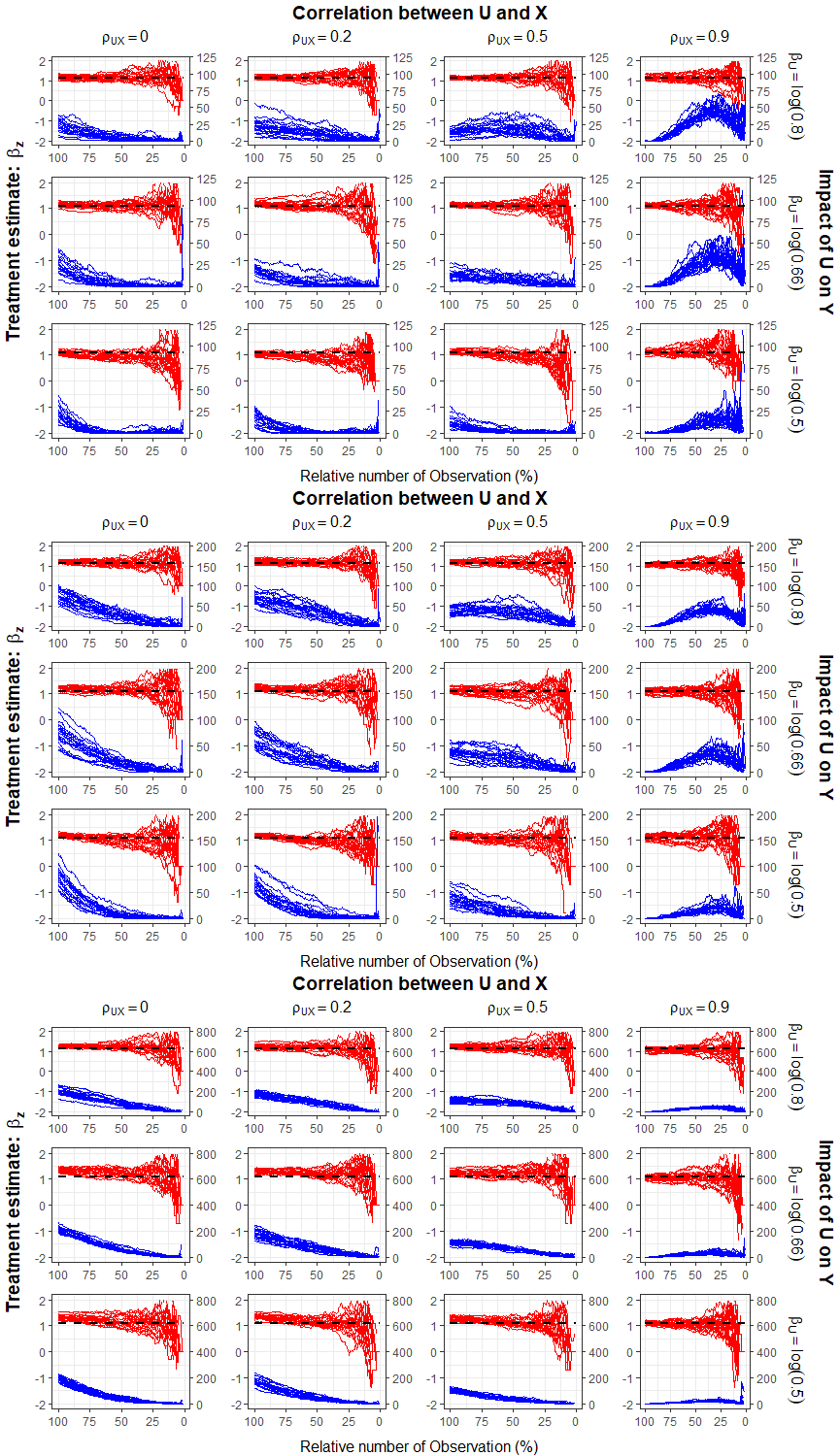


**Fig. S8:** Trajectories of treatment effect (left y-axis, red) on the log(HR) scale and sum of squared z-differences (right y-axis, blue) for balance measuring of the omitted covariate $U$ for the 500 simulated data sets. Dashed black lines show the true, conditional treatment estimate$\beta_{z}= log \left( 3 \right)$. All scenarios assume the omission of a true confounder $U$ with **A**: low negative impact on treatment allocation, i.e., $\alpha_{u}=\log(0.8)$ **B**: moderate negative impact on treatment allocation, i.e., $\alpha_{U}=\log\left( 0.66 \right)$. **C**: high negative impact on treatment allocation, i.e. $\alpha_{U}=\log\left( 0.5 \right)$


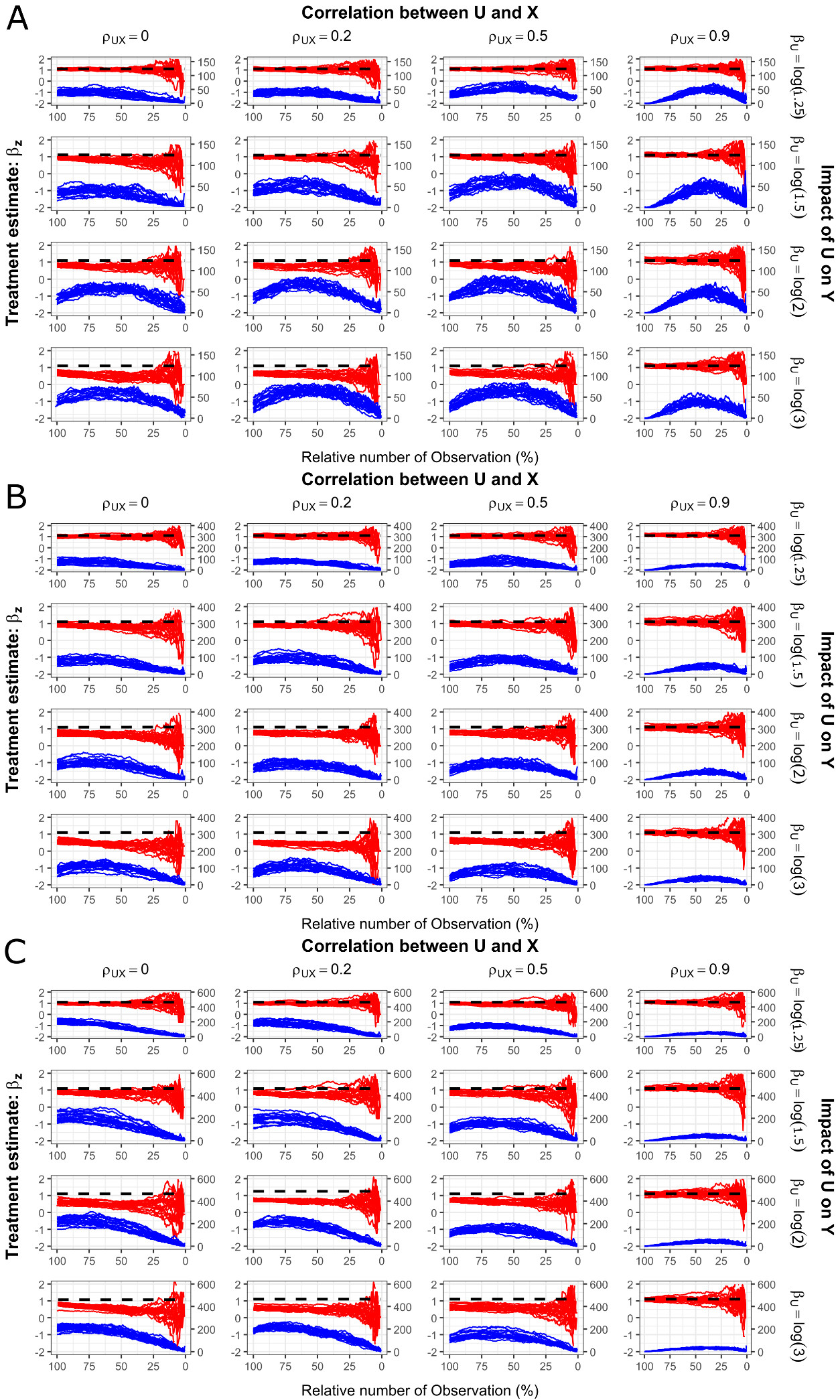


**Fig. S9:** Trajectories of treatment effect (left y-axis, red) on the log(HR) scale and sum of squared z-differences (right y-axis, blue) for balance measuring of the omitted covariate $U$ for the 500 simulated data sets. Dashed black lines show the true, conditional treatment estimate$\beta_{z}= log \left( 3 \right)$. All scenarios assume the omission of a true confounder $U$ with **A**: low negative impact on treatment allocation, i.e., $\alpha_{u}=\log(0.8)$ **B**: moderate negative impact on treatment allocation, i.e., $\alpha_{U}=\log\left( 0.66 \right)$. **C**: high negative impact on treatment allocation, i.e. $\alpha_{U}=\log\left( 0.5 \right)$


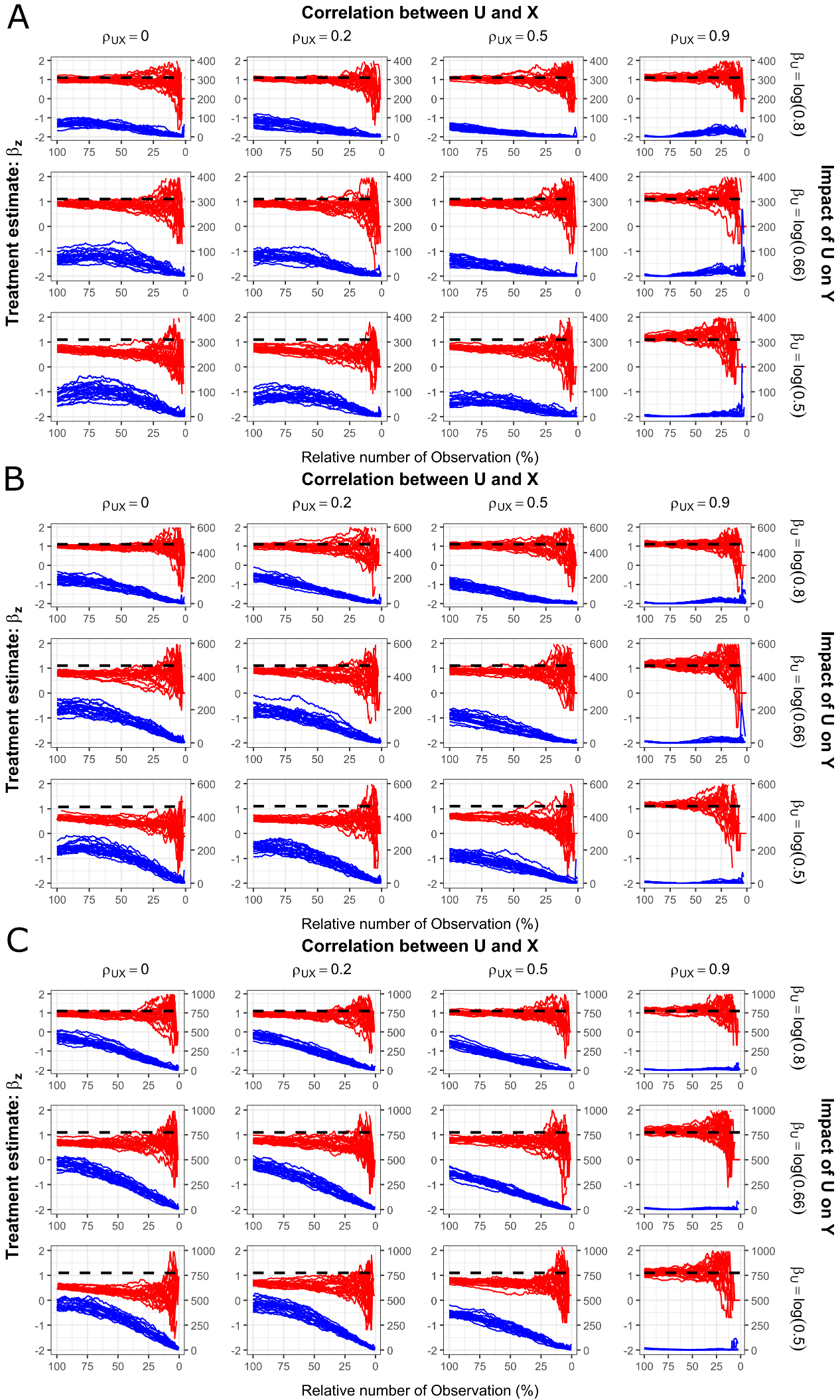


**Fig. S10:** Trajectories of treatment effect (left y-axis, red) on the log(HR) scale and sum of squared z-differences (right y-axis, blue) for balance measuring of the omitted covariate $U$ for the 500 simulated data sets. Dashed black lines show the true, conditional treatment estimate$\beta_{z}= log \left( 3 \right)$. All scenarios assume the omission of a true confounder $U$ with **A**: low positiv impact on treatment allocation, i.e., $\alpha_{u}=\log(1.25)$ **B**: moderate positv impact on treatment allocation, i.e., $\alpha_{U}=\log\left( 2 \right)$. **C**: high postiv impact on treatment allocation, i.e. $\alpha_{U}=\log\left( 3 \right)$


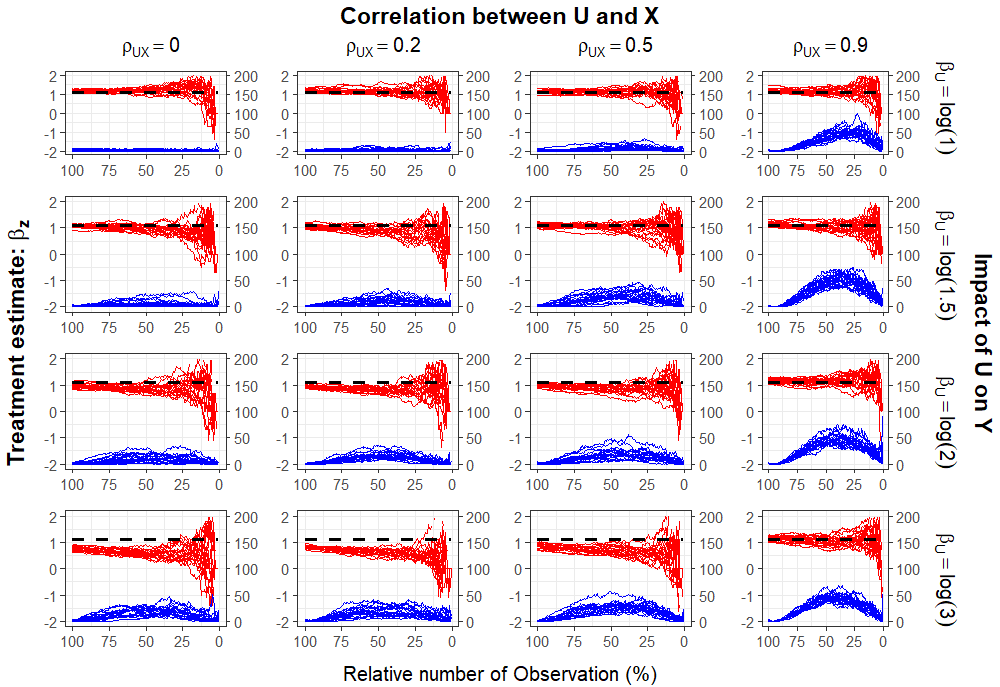


**Fig. S11:** Trajectories of treatment effect (left y-axis, red) on the log(HR) scale and sum of squared z-differences (right y-axis, blue) for balance measuring of the omitted covariate $U$ for the 500 simulated data sets. Dashed black lines show the true, conditional treatment effect estimate$\beta_{Z}=\log\left( 3 \right)$. First row shows results for the omission of an instrumental variable, all other rows show the results for the omission of a prognostic factor.

1. Please note that in the present manuscript, we address the special case of a Cox model that includes only treatment (no adjustments are made). However, if a Cox model is assumed for three prognostic factors (e.g., one treatment and two additional prognostic factors), then a Cox model conditioned on the treatment and one of the additional prognostic factors is also referred to as a "marginal" Cox model (more precisely, marginal with respect to the omitted third prognostic factor), providing a marginal treatment estimate even though the Cox model is fitted for one prognostic factor. [↑](#footnote-ref-1)
